# *BZcon1*, a SANT/Myb-Type Gene Involved in the Conidiation of *Cochliobolus carbonum*

**DOI:** 10.1534/g3.114.012286

**Published:** 2014-06-03

**Authors:** Jun-xiang Zhang, Yi-xin Wu, Honhing Ho, Hao Zhang, Peng-fei He, Yue-qiu He

**Affiliations:** * Faculty of Plant Protection, Yunnan Agricultural University, Kunming 650201, Yunnan, China; † Faculty of Agriculture and Biotechnology, Yunnan Agricultural University, Kunming 650201, Yunnan, China; ‡ Department of Biology, State University of New York, New Paltz, New York 12561

**Keywords:** *Bipolaris zeicola*, conidia, sporulation, clone, Northern Leaf Spot

## Abstract

The fungal pathogen *Cochliobolus carbonum* (anamorph, *Bipolaris zeicola*) causes Northern Leaf Spot, leading to a ubiquitous and devastating foliar disease of corn in Yunnan Province, China. Asexual spores (conidia) play a major role in both epidemics and pathogenesis of Northern Leaf Spot, but the molecular mechanism of conidiation in *C. carbonum* has remained elusive. Here, using a map-based cloning strategy, we cloned a single dominant gene, designated as *BZcon1* (for *Bipolaris zeicola* conidiation), which encodes a predicted unknown protein containing 402 amino acids, with two common conserved SANT/Myb domains in N-terminal. The *BZcon1* knockout mutant completely lost the capability to produce conidiophores and conidia but displayed no effect on hyphal growth and sexual reproduction. The introduced *BZcon1* gene fully complemented the *BZcon1* null mutation, restoring the capability for sporulation. These data suggested that the *BZcon1* gene is essential for the conidiation of *C. carbonum*.

In many phytopathogenic fungi, asexual sporulation in the form of conidia is critical in the life cycle, and so deciphering the molecular mechanisms involved in conidiation development is a prerequisite to provide novel strategies for disease management. The survival of these fungi is ensured by the production and dissemination of spores that are long-lived and highly resistant to environmental stresses. Spore germination represents a critical stage in the life cycle of fungi and constitutes a prerequisite for colonization in a new environment (Lafon *et al.* 2005). The process of conidiation involves many common developmental themes, including gene expression, cell specialization, and intercellular communication. For example, in *Aspergillus nidulans* conidiation, three central regulatory genes (*brlA*, *abaA*, and *wetA*) control conidiation-specific gene expression and determine the order of gene activation during conidiophore development and conidial maturation (Park and Yu 2012). These studies suggest that it is important to develop new strategies to control the occurrence of fungal disease by deciphering the molecular mechanism involved in asexual sporulation of plant pathogenic fungi.

Asexual spores play a major role in the epidemics of *Cochliobolus carbonum* (anamorph, *Bipolaris zeicola*), which causes Northern Leaf Spot, a ubiquitous and devastating foliar disease of corn in many regions of the world (Nelson 1959, 1970; Welz and Leonard 1994; Zhang *et al.* 2013). In the wild, *C. carbonum* exists in the asexual form, reproducing mainly through the production of conidia. As soon as the conidia are in contact with the corn surface, they release a copious, two-layered, extracellular matrix (ECM) resulting in the adhesion of germlings of the fungus to the host surface (Evans *et al.* 1982; Apoga *et al.* 2001). After penetration of the germ tubes, infectious hyphae initially grow within the plant cells. Eventually, the plant cells are killed and thousands of conidia could be released from each lesion. Despite the importance of conidia in pathogenic epidemics, little is known about the molecular mechanism of conidiation in *C. carbonum*.

In ascomycetes, conidia formation requires the temporal and spatial control of cell differentiation, which is a process under polygenic control (Adams *et al.* 1998; Etxebeste *et al.* 2010). To date, no genes involved in conidiation have been reported in the asexual development of *C. carbonum*. Therefore, it would be valuable to identify and characterize genes that are specifically involved in conidia formation for an in-depth understanding of the asexual development. Development of genetic maps and the identification of molecular markers closely linked to target genes have allowed the map-based cloning of specificity genes governing the phenotype (Sweigard *et al.* 1995; Farman and Leong 1998; Ma *et al.* 2006). Here, via both a map-based cloning strategy and functional experiments, this study characterizes a novel *C. carbonum* gene, *BZcon1*, which is a determinant of conidiation in this fungus. *BZcon1* encodes a potential binding protein containing 402 amino acids as putative transcription factors and regulates the conidiation in *C. carbonum*.

## Materials and Methods

### Fungal strains, cultures, and transformation

Field isolates S92 and S129 were collected from corn plants with Northern Leaf Spot symptoms in Yunnan Province, China, in 2010. Experimental strains Aa82, Aa108, and Aa113 were obtained from the progeny of the cross S92 × S129. Other strains in this study are shown in Table 1. Mating types of all isolates were determined by polymerase chain reaction (PCR) amplification as described previously by Gafur *et al.* (1997). Mycelia collected from potato dextrose agar (PDA) plates were used for isolating fungal genomic DNA and RNA. Mycelia collected from Fries medium (Fries 1978) were used for obtaining the protoplasts. Protoplasts were isolated and transformed as described by Liu and Friesen (2012). The selection medium was supplemented with 100 µg/ml hygromycin B (Roche, USA) or 500 µg/ml G418 (Ameresco, USA) for the screening for hygromycin B–resistant (HR) or G418-resistant (GR) transformants, respectively. Single conidium of the transformant was isolated to ensure nuclear homogeneity (Choi *et al.* 1999). If lack of conidia precluded single-spore isolation, then transformants were transferred three to four times to select against heterokaryons (Lev *et al.* 1999). Isolates and mutants were stored on filter paper disks as previously described (Silué *et al.* 1992).

**Table 1:** Wild-type and mutant strains of *Cochliobolus carbonum* used in this study

Strains	Brief Description	Growth (cm)*^a^*	Conidiation (×10^4^)*^b^*
S92	Field isolate, *MAT1-2-1*	7.52 ± 0.10*^c^*	101.34 ± 0.0*^c^*
S129	Field isolate, *MAT1-1-1*	7.55 ± 0.21	0
Aa82	Experimental isolate, *MAT1-2-1*	7.62 ± 0.04	120.12 ± 0.04
Aa108	Experimental isolate, *MAT1-1-1*	7.62 ± 0.03	110.11 ± 0.06
Aa113	Experimental isolate, *MAT1-2-1*	7.53 ± 0.12	110.23 ± 0.03
M113-1	*BZcon1* knockout mutant of Aa113	7.56 ± 0.02	0
C113-2	*BZcon1* complemented transformant of M113-1	7.51 ± 0.06	12.13 ± 0.04
C129-1	*BZcon1* complemented transformant of S129	7.54 ± 0.02	10.25 ± 0.06

a Vegetative growth was measured at day 6 after incubation on PDA plate.

b Conidiation was measured by counting the number of conidia collected with 10 ml of sterilized distilled water from 6-cm diam. PDA plate at day 10 after incubation at 25°.

c Data were presented as mean and SDs were calculated from four independent experiments.

### Cross and conidiation assay

The isolates S92, Aa82, and Aa113 of *C. carbonum* produced abundant conidia (con^Y^), whereas the isolate S129 did not produce conidia (con^N^) on PDA plates. Crossing was performed according to the description of Welz and Leonard (1994) on Sachs agar medium (Gafur *et al.* 1998). Pseudothecia were harvested 20 to 25 days after crossing, and single ascospores were isolated from the asci and allowed to germinate on PDA. For conidiation assays, all isolates were incubated on PDA plates (6-cm diameter) at 25° for 10 d in the dark. Then, the colony surface was washed with 10 ml sterile distilled water (SDW) and the conidia suspension was filtered through two layers of cheesecloth. The amount of conidia per milliliter of the suspension was determined by counting the conidia with a hemacytometer using light microscopy. An isolate was regarded as a con^Y^ or a con^N^ progeny with the presence or absence of conidia, respectively. Each isolate was tested at least four times. Segregation of con^Y^ in the progeny population was examined with a χ^2^ test for goodness of fit for con^Y^/con^N^ ratios. A summary of the segregation populations is shown in Table 2.

**Table 2:** Segregation of random ascospore progeny from crosses between conidia-producing isolates (con^Y^) and isolates producing no conidia (con^N^) of *Cochliobolus carbonum*

Cross Code	Cross (con^Y^×con^N^)	Generation	No. of Progeny		Expected Ratio	χ^2^	Probability
			Con^Y^	Con^N^			
Aa	S92×S129	F1	158	150	1:1	0.20	0.65
BC	Aa82×S129	Backcross	26	24	1:1	0.08	0.78
DD	Aa113×S129	Backcross	134	137	1:1	0.03	0.86
KC	Aa108×M113-1	—	59	55	1:1	0.14	0.71

### Linkage marker screening of the con^Y^ locus

Pools of DNA for bulked segregant analysis (BSA) (Michelmore *et al.* 1991) were prepared by combining equal amounts of DNA from five con^Y^ or five con^N^ progeny strains. RAPD (Welsh and McClelland 1990; Williams *et al.* 1990), ISSR (Zietkiewicz *et al.* 1994), and SRAP (Li and Quiros 2001) amplifications were performed on each pool and parental strains. RAPD reaction productions were analyzed by electrophoresis on agarose gels (1.2%). The specific fragment for con^Y^ pools was excised from the agarose gels. ISSR and SRAP reaction productions were resolved in polyacrylamide gels (6%) coupled with silver-staining. The exclusive band for con^N^ pools was excised from polyacrylamide gels as described by Zhang *et al.* (2009). The PCR production was excised and purified using the MinElute Gel Extraction Kit (Sangon Biotech, China). The eluted fragment was ligated into pMD18-T simple vector (Takara, Japan) following the supplier’s instructions and transformed into competent *Escherichia coli* TG1, and then sequenced by Beijing Genomics Institute (BGI; Beijing, China). The sequence obtained was used to design primers for sequence characterized amplified regions (SCARs) (Paran and Michelmore 1993) analysis. The successful SCAR primer sequences used are shown in Table 3.

**Table 3:** List of oligonucleotides used in this study

Name	Sequence (5′-3′)*^a^*
SC254	F, TGGGTCCCCAGCAAACCG
	R, TGGGTCCCCCTGCTTAGC
SC383	F, TGGGTCCCTCTTACCTTTTG
	R, TGGGTCCCTCACAATATGCC
SC403	F, GGGGGATGAGGCGGGTAGAG
	R, GGGGGATGAGACCGATAACAGT
SC829	F, GTGTGTGTGTGTCGGATGG
	R, GTGTGTGTGTGGCCTGAATCAA
SC855	F, ACACACACACACCTGGCAC
	R, ACACACACACACACACCTCCTT
SC24	F, GAATTTGCCCGCATTTTTCC
	R, GTCCAAACCGGACCCTGAAC
S8-35	F, AAGCTTGGGTTCTTACGCGA
	R, CCTCGAATGGACCCGAC
S8-33	F, AAGCTTATCGGCGAACTGC
	R, CGTGATGGAGCACGCGA
S1-125	F, AAGCTTCTCCCGGAAGCTATCA
	R, GTAAGTTCTCCCTTCACTCAAGATT
S1-123	F, AAGCTTTGTCAATAGAATCCTTTTTGG
	R, CATCCCCTCACTTGTAAATACACAT
SSR2	F, TGCTATTGTCGTCGCCGC
	R, ATTTTATTTTATTTTA
CF1	ATGGTTGCCCACAGACG
CR1	CTACATGATGTTTCGCAGAG
C-F1	CCGCTCGAGAGAACGCTG
C-R1	AATAAGCTTGGGCCCTGAGCAGCAAAA
C-F2	AATAAGCTTAATAGCCGAGTTGTCCACCA
C-R2	AAAGAATTCTAGCAACGGCATAGCAACA
HB-CF1	AAAGGATCCGCCGAAACGAGAACAAGATG
HB-CR1	AAAGGATCCTCGAGAGAACGCTGCCT
H-F2	ATGAAAAAGCCTGAACTCAC
H-R2	CTATTCCTTTGCCCCTCG

F, forward primer; R. reverse primer.

a The sequence underlined indicates the restriction cut site for construction of knockout and complementation vector.

### Genetic mapping of the gene *BZcon1*

For genetic linkage analysis, SCAR amplifications were performed with genomic DNA from 271 progenies of the cross between Aa113 and S129. Segregation data obtained were analyzed with the software Mapmaker version 3 (Lander *et al.* 1987). Parameters for map construction were a minimum logarithm of odds (LOD) score of 3.0 and a maximum recombination fraction of 0.4. The Kosambi function was used to compute the recombination distances in centimorgans (cM).

### Construction and screening of BAC library

The Aa113 genomic DNA bacterial artificial chromosome (BAC) library was constructed as described by Brosch *et al.* (1998) with minor modifications. After *Hind*III partial digestion, DNA fragments 75 to 120 kb in size were ligated into plasmid pBeloBAC11 and electroporated into *E. coli* TG1 using an electroporator (Eppendorf, Germany). After electroporation, the cells were resuspended in 600 µl of LB medium (10 g tryptone, 5 g yeast extract, and 10 g NaCl in 1000 ml deionized water), allowed to recover for 1 h at 37° with gentle shaking, and then plated on LB agar containing chloramphenicol (12.5 μg/ml). The plates were incubated overnight, and the recombinants were picked manually and transferred to 96-well plates that were incubated for 10 h with shaking at 220 rpm. The BAC library was stored at −80°. Screen of the BAC clone by pool method was first performed with the markers SC403 and SC254. Gaps present in the assembled sequence were further filled as described by Zhou *et al.* (2006). The selected BAC clones were sequenced, and the obtained sequences were assembled using the Bioedit software (http://www.mbio.ncsu.edu/bioedit/bioedit.html).

### Chromosome walk and gene prediction

We adopted chromosome walking of physical analyses to delimit the location of the *BZcon1* gene. For the first move, we constructed BAC contig mapping and confined gene *BZcon1* to a genomic interval between markers SC403 and SC254, and then this interval was analyzed using SSR finder (http://www.fresnostate.edu/ssrfinder). For the second move, obtained SSR sites were used to design primers detecting polymorphism between the parents, and the screened primers were further used to amplify the special fragment of genomic DNA from 271 progenies of the cross between Aa113 and S129, respectively. A marker, SSR2 (Table 3), developed based on SSR-PCR amplification, further delimited *BZcon1* to a 54,526-bp genomic interval. Genes of the derived interval sequence were predicted using the Fgenesh program (http://linux1.softberry.com/berry.phtml).

### Nucleic acid manipulation

Total DNA of the isolates and mutants was extracted and purified from the mycelium by using the cetyltrimethylammonium bromide (CTAB) protocol (He 2000). PCR amplifications were performed in a total volume of 20 μl containing 0.4 µM of each dNTP, 5 µM of each primer, 1 unit of easy*Taq* or 2 units easy*pfu* DNA polymerase (Trans, China), 2.0 µl of 10× reaction buffer, and 10 to 20 ng of genomic DNA. Total RNA was extracted from the mycelia grown for 3 to 8 d on the PDA plates using the Trizol reagent (Invitrogen, USA) according to the manufacturer’s protocol. Reverse-transcription PCR (RT-PCR) was performed with the PrimeScript strand cDNA Synthesis Kit (Takara, Japan) following the supplier’s instructions. Procedures for gel electrophoresis, plasmid DNA preparations, and restriction enzyme digestions were performed following standard protocols (Smith 1993; Stacey and Isaac 1994). For Southern blot analysis, genomic DNA was isolated from *C. carbonum* wild-type strain Aa113 and putative *BZcon1* deletion mutant M113-1. Aliquots of 15 mg DNA were digested with *Sal*I, separated by electrophoresis in 0.8% agarose gels, and transferred onto a Hybond N^+^ membrane (Amersham Pharmacia Biotech). The probe was amplified with primers H-F2 and H-R2 (Figure 2A and Table 3), whereas probe labeling and hybridization were performed with Amersham ECL Direct Nucleic Acid Labeling and Detection system (GE Healthcare UK Limited Companies), following the manufacturer’s instructions. All primers (Table 3) were synthesized by BGI.

### Knockout of gene *BZcon1*

For *BZcon1* knockout, the replacement vector (pBSKC1) favoring double-crossover integration was constructed in three steps (Figure 2A). First, a 1084-bp *Xho*I-*Hind*III upstream fragment of the *BZcon1* gene was ligated to *Xho*I-*Hind*III–digested pBlueScript II KS (+) (pBS), and the ligated plasmid was denominated as pBSP11. Then, a 1317-bp *Hind*III-*Pst*I downstream fragment of the *BZcon1* gene was ligated to the *Hind*III-*Pst*I–digested pBSP11, and the ligated plasmid was designated as pBSP11P12. Subsequently, a 2.0-kb hygB resistance gene cassette (*hph*), digested with *Hind*III from plasmid pTFCM (Li *et al.* 2005), was ligated to *Hind*III-digested pBSP11P12. Homologous fragments used in the construction of the knockout vector were amplified using Aa113 as the template with two pairs of primers, C-F1/C-R1 and C-F2/C-R2, respectively (Table 3). Circular pBSKC1 was transformed into Aa113, and targeted integration of the plasmid into the *C. carbonum* genome was confirmed by DNA gel blot analysis. The transformants were further determined by PCR with the primer pairs CF1/CF2 (Table 3).

### Complementation of gene *BZcon1*

For complementation assays, a *BZcon1* region including the *BZcon1* along with its 1.7-kb upstream and 0.4-kb downstream sequences was amplified using Aa113 as the template with primer pair HB-CF1/HB-CR1 (Table 3). It was subsequently cloned into the *BamH*I sites in plasmid pFA6a-kanMX6 harboring a G418 resistance cassette (KanMX) as pFACON31. Circular pFACON31 was transformed into the *BZcon1* mutant M113-1.

## Results

### Map-based cloning of the gene *BZcon1*

The progeny resulting from three independent crosses segregating for conidiation was summarized in Table 2. The segregation of conidial and nonconidial strains in the F1 progeny (cross S92/S129) and BC populations (Aa82/S129 and Aa113/S129) fitted a ratio of 1:1 ratio in the χ^2^ test of goodness-of-fit, indicating that the asexual sporulation of *C. carbonum* is controlled by a single dominant gene. We designated this gene as *BZcon1* (for *Bipolaris zeicola* conidiation).

**Figure 1:**
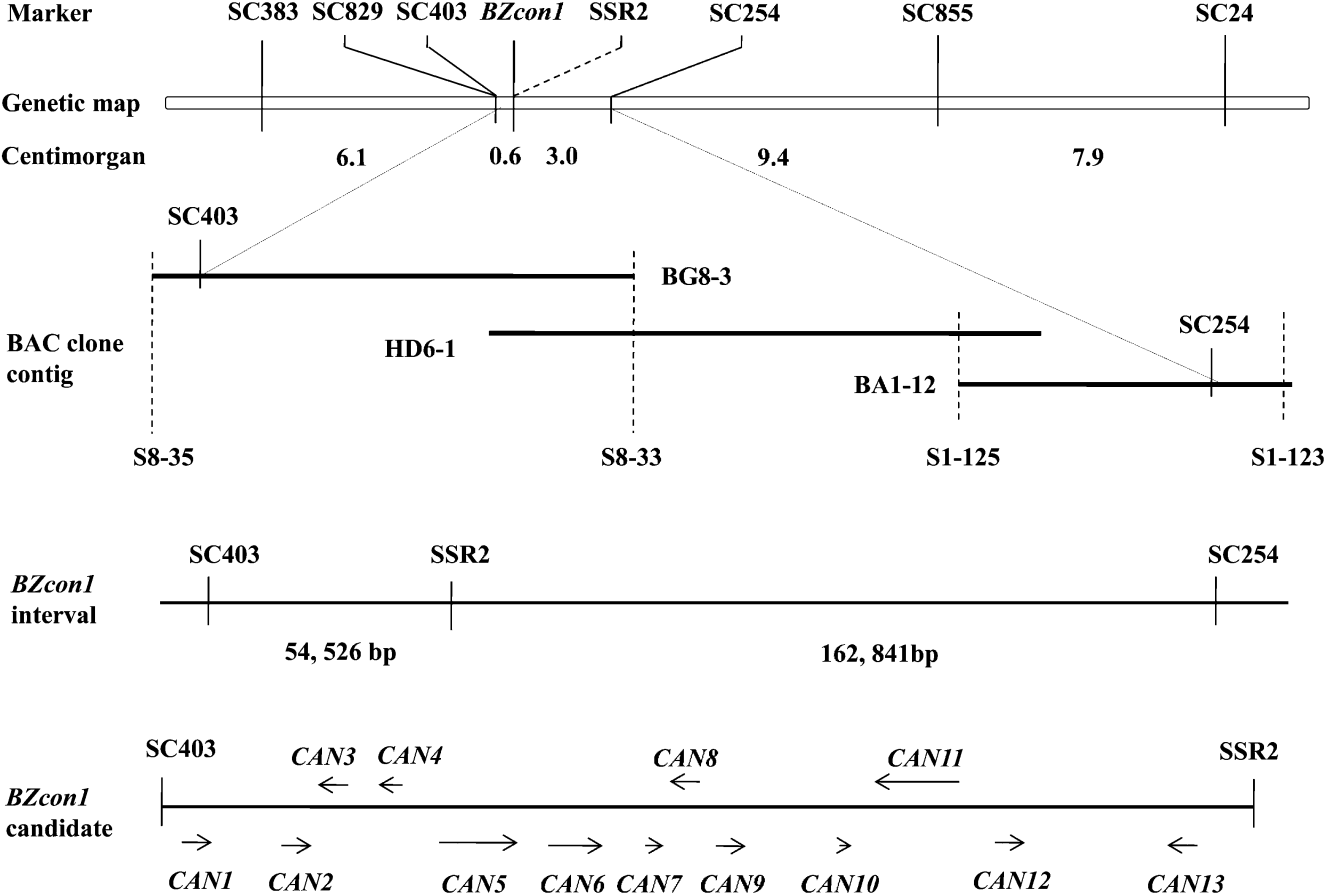
Genetic and physical maps around the *BZcon1* locus in the isolate Aa113. Genetic maps were constructed using multipoint linkage analysis on segregating populations of 271 progeny with the Mapmaker/Exp 3.0 software. Dashed lines connect the physical location of markers to their location on the genetic map or connect markers to their location in the bacterial artificial chromosome (BAC) clone contig. The gene was predicted according to Fgenesh programs and the arrows correspond to position of gene candidates. *CAN13* is the gene identified as a candidate for *BZcon1* and used for knockout and complementation assays.

A PCR-based marker system was used to detect polymorphism between two parental isolates. Using a BC family of 271 strains produced by crossing the con^Y^ isolate Aa113 and con^N^ isolate S129, a total of six SCAR primers were found to be linked to the targeted *BZcon1* gene. By using software MapMaker 3.0, the *BZcon1* locus was mapped between SC403 and SC254, which were identified as 0.6 and 3.0 cM, respectively (Figure 1).

The construction of a BAC contig including the *BZcon1* locus in con^Y^ parental isolate Aa113 was initiated by SC403 and SC254 markers. By PCR screening, SC403 and SC254 marker fragments were detected in BAC clones BG8-3 and BA1-12, respectively. So, BG8-3 and BA1-12 were completely sequenced and a gap was present between BG8-3 and BA1-12. The BG8-3 and BA1-12 end sequences were then used to design several contiguous markers (Figure 1 and Table 3) and allowed the identification of a BAC clone (HD6-1) bridging the gap. HD6-1 was also completely sequenced. As a result, the gene *BZcon1* interval, between markers SC403 and SC254, consisted of three overlapping BAC contigs, which covered a distance of 217,367 bp (Figure 1), giving an average physical-to-genetic-distance ratio of 60.4 kb/cM.

Forty-four SSR sites were obtained from the *BZcon1* interval sequence using SSR finder. The SSR-PCR amplifications showed that only marker SSR2 was identified as co-segregation with *BZcon1*. By using MapMaker 3.0 software, the *BZcon1* locus was determined, located further between markers SC403 and SSR2, and the physical distance between SC403 and SSR2 was 54,526 bp. A total of 13 genes, designated *CAN1* to *CAN13*, were predicted in this region (Figure 1).

These genes were used to design gene code–specific primers (data not shown) and applied to the amplification of con^N^ isolate S129. The obtained sequences were determined. It was found that the coding sequences in the con^Y^ isolate Aa113 were identical to the ones in con^N^ isolate S129, indicating that the transcriptions of genes may be different. RT-PCR experiments showed *CAN2*, *CAN10*, and *CAN1*2 were not detected in both of these two strains, and *CAN1*, *CAN3*, *CAN4*, *CAN5*, *CAN6*, *CAN7*, *CAN8*, *CAN9*, and *CAN11* were both present in Aa113 and S129. The remaining *CAN13* was conspicuously absent in the con^N^ isolate S129 in RT-PCR experiments, showing that *CAN13* could be the only *BZcon1* gene candidate.

### Determination of *BZcon1* by gene knockout and complementation tests

**Figure 2:**
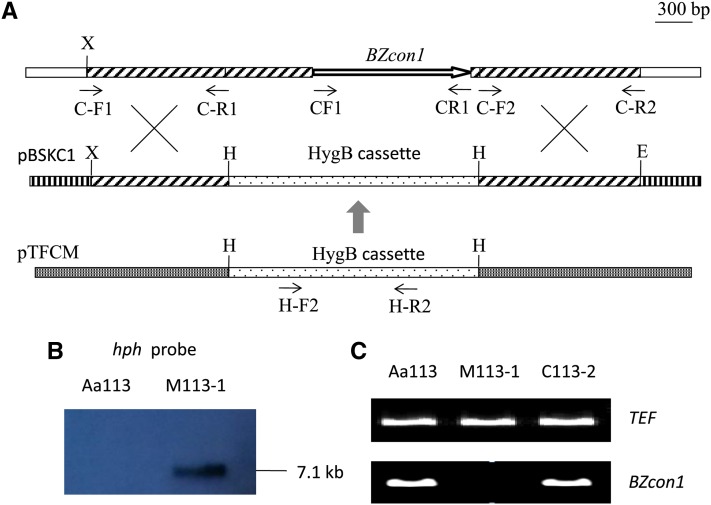
Strategy for *BZcon1* gene knockout and confirmation of *BZcon1* mutant. (A) *BZcon1* knockout vector pBSKC1 contains a 2.0-kb hygB resistance gene cassette. Homologous recombination through a double crossover event results in the knockout of a *BZcon1* region with the hygB resistance cassette (*hph*). This region includes a 634-bp-long upstream fragment, the entire open reading frame (1209-bp-long) of *BZcon1* gene, and a 76-bp-long downstream fragment. The positions of primers are indicated by small arrows. X, *Xho*I. H, *Hind*III. E, *EcoR*I. (B) Southern blot analysis. Total genomic DNA samples isolated from Aa113 (experimental isolate) and M113-1 (*BZcon1* knockout mutant of Aa113) were digested with *Sal*I. The *hph* prober, a 1-kb PCR fragment amplified from pBSKC1 using primers H-F2 and H-R2, is exactly the *BZcon1* region replaced by the hygB resistance gene. (C) Total RNA extracted from the mycelia of Aa113, M113-1, and C113-2 grown for 3 to 8 d on PDA plates, respectively. And obtained RNA was subjected to RT-PCR using *BZcon1* gene-specific primers CF1 and CR1. The RT-PCR product is an approximately 1.2-kb fragment in Aa113 and C113-1 (*BZcon1* complemented transformant of M113-1) as predicted, but is missing in M113-1.

**Figure 3:**
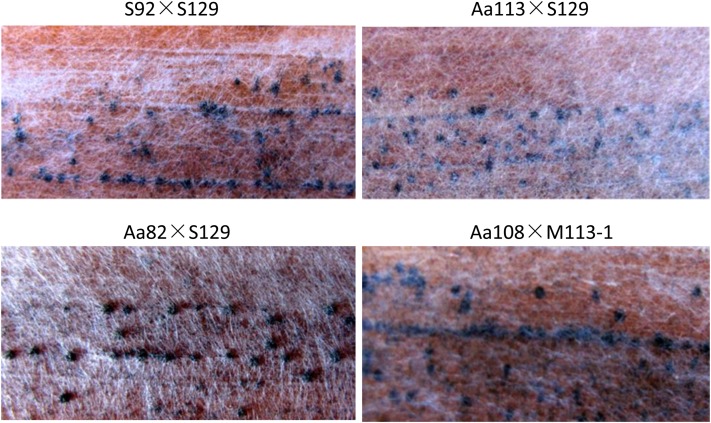
Mating result of the *BZcon1* knocked-out mutant on Sachs agar medium. Pseudothecia is visible as dark spots. S92, field isolate, *MAT1-2-1*; S129, field isolate, *MAT1-1-1*; Aa113, experimental isolate, *MAT1-2-1*; Aa82, experimental isolate, *MAT1-2-1*; Aa108, experimental isolate, *MAT1-1-1*; and M113-1, *MAT1-2-1*, *BZcon1* knockout mutant of Aa113.

To assess whether *CAN13*, as *BZcon1* gene candidate, is responsible for the conidiation of the fungus, homologous recombination was used to replace *CAN13* with the plasmid pBSKC1, which contains the 2.0-kb hygB resistance cassette. The wild-type strain Aa113 was transformed with pBSKC1, and 26 HR transformants were isolated. All the transformants did not form conidia on the PDA. Genetic crosses were performed between HR transformants, the mutant M113-1 and an experimental strain, Aa108 (Figure 3). We then isolated 114 single ascospore strains from the progeny of this cross. All 55 HR strains failed to produce conidia, but the 59 hygromycin-sensitive (HS) strains were normal in conidia production. PCR amplification with CF1 and CR1 revealed that all HS strains had an approximately 1.2-kb band that was absent in all HR strains. This proves to be only one locus replaced by the hygB resistance gene. Cosegregation of the replacement event and HygB resistance was also confirmed by Southern bolt analysis (Figure 2B).

**Figure 4:**
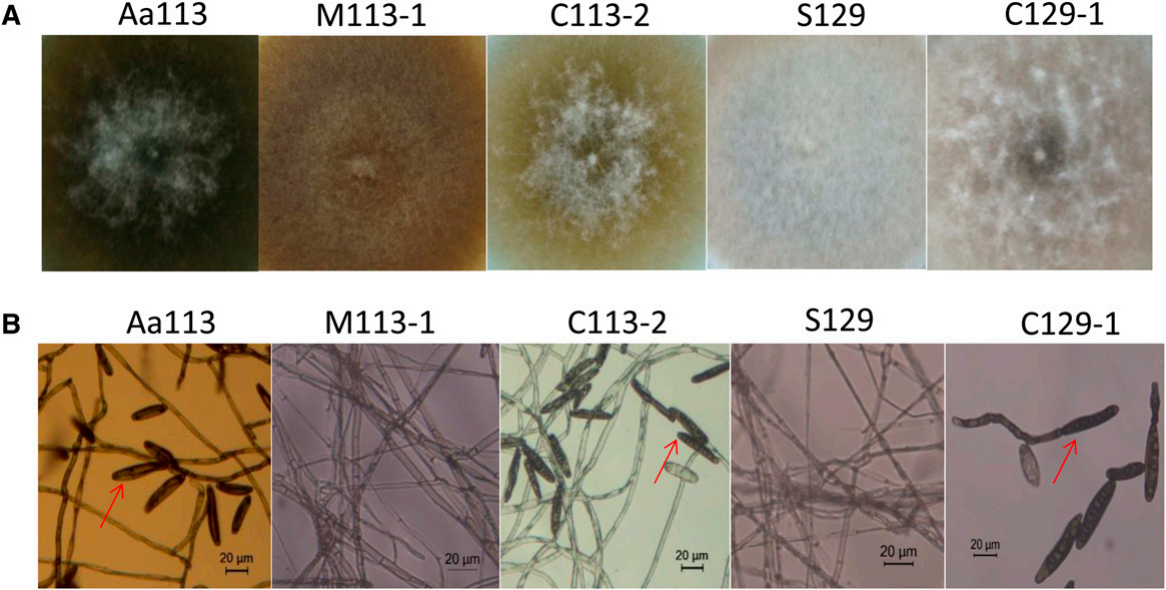
Phenotypes of the wild-type strain Aa113 and S129, *BZcon1* knockout mutant M113-1, *BZcon1* complemented transformants C113-2 and C129-1. (A) Colony morphology of Aa113, S129, M113-1, C113-2, and C129-1 on potato dextrose agar (PDA) plates inoculated for 6 d. (B) Plate cultures observed under light microscope (red arrows showing conidia).

To further confirm that the disruption of the *CAN13* was responsible for the phenotype of the *CAN13* mutant, we conducted a complementation assay by transferring the vector pFACON31 harboring the full-length *CAN13* and a G418-resistant gene as a selectable marker into the mutant, M113-1. One G418-resistant complementary transformant, C113-2, fully complemented the *CAN13* null mutation, restoring the capability for sporulation (Table 1 and Figure 4B). Likewise, the vector pFACON31 was introduced into the con^N^ strain S129, and the transformant, C129-1, gained the capability to produce conidia on PDA. Additionally, based on RT-PCR analysis, *CAN13* transcripts were detected in Aa113 and C113-2, but were absent in M113-1 (Figure 2C). These data clearly demonstrated that *CAN13* and *BZcon1* was the same gene.

### *BZcon1* is required for conidiation

After growing on PDA plates for 6 d, all colony diameters were determined by using at least four replicates for each strain or mutant. The *BZcon1* deletion mutant, M113-1, displayed no obvious defects in the mycelial growth rate when compared with the strain Aa113 (Table 1), but M113-1 produced gray–white aerial hyphae similar to that of the S129, in contrast to Aa113, which had gray–green aerial hyphae (Figure 4A). In sporulation assays, M113-1 completely lost the capability to produce conidia on PDA. When the aerial hyphae were examined, abundant conidiophores and conidia were produced by Aa113, but none at all were produced by M113-1 mutant. These results indicated that the *BZcon1* was essential for conidiation of *C. carbonum*, and the development of conidia and conidiophores was blocked in the mutant (Figure 4B).

### A short upstream promoter region potentially mediates transcriptional regulation of the gene *BZcon1*

**Figure 5:**
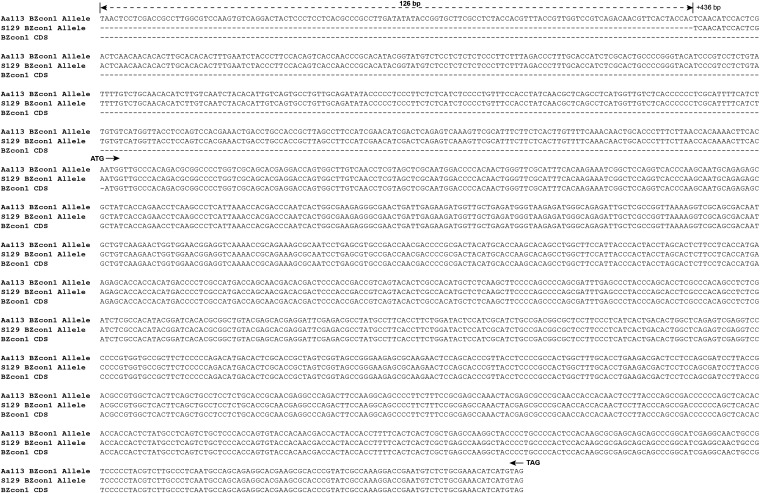
Sequence comparisons of the upstream region of *BZcon1* from two isolates A113 and S129. Dot line with double arrows shows the absence of 126 continuous nucleotides in the upstream (+426 bp) of *BZcon1* from the isolate S129 when compared with that of Aa113.

Approximately 1.7-kb upstream sequences of *BZcon1* from the isolates S129 (not produce conidia in PDA; without transcription of *BZcon1*) were amplified and sequenced. Sequence alignment found the absence of 126 continuous nucleotides in the upstream of *BZcon1* from the isolate S129 when compared with that of Aa113 (Figure 5). We further observed that transformant C129-1 can gain the ability to produce conidia on PDA via acquiring a segment including *BZcon1* with the 1.7-kb upstream sequence of Aa113. The results indicated that the deficiency of a short putative promoter 126 bp in length may contribute to limitation of *BZcon1* transcription.

### *BZcon1* encodes a predicted unknown protein with two conserved SANT/Myb-like domains at N terminal

**Figure 6:**
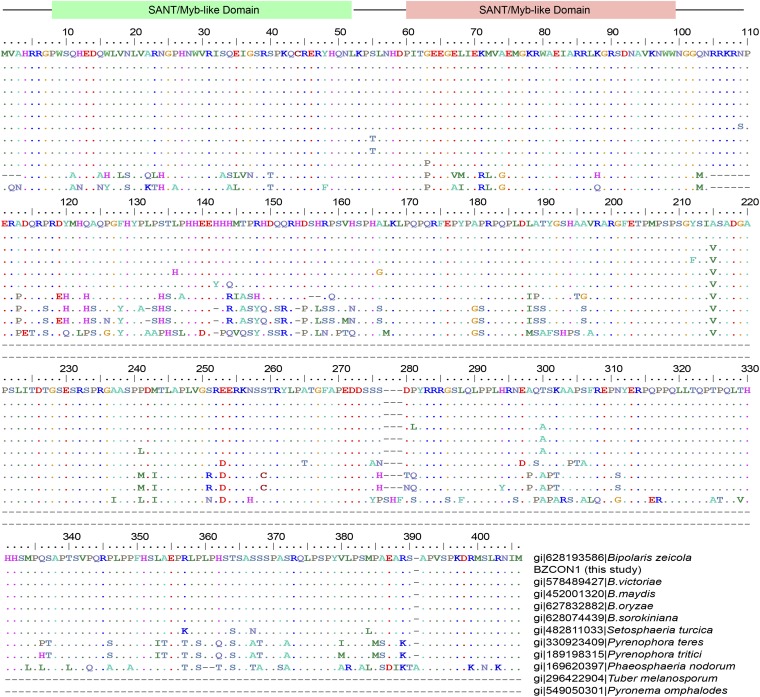
An alignment of the amino acid sequences of BZCON1 and its 11 related homologs. Two SANT/Myb-like domains were located at N-terminal (approximately 100 amino acids in length).

The ORF of *BZcon1* gene (GenBank accession number: KJ562713) was 1209 bp long, encoding 402 amino acids, and there was no intron in the ORF. To confirm the absence of introns, a full-length *BZcon1* complementary DNA (cDNA) was obtained from Aa113 by RT-PCR using primers CF1/CR1. The amino acid sequence of *BZcon1* was analyzed using InterProScan (Hunter *et al.* 2012), showing *BZcon1* has two SANT/Myb domains in the N-terminal region (approximately 100 amino acids in length). This result indicated that *BZcon1* should be a putative Myb-like transcription factor. To further strengthen this prediction, using amino acid sequence of BZCON1, we performed blast searches (E-value <1.00e-5) against nonredundant protein sequence database (ftp://ftp.ncbi.nlm.nih.gov/blast/db/) and revealed that all 164 top-blast hits were derived from two related fungal phylum Ascomycota (162 hits) and Basidiomycota (2 hits) (Supporting Information, Table S1 and Figure S1), having two common conserved SANT/Myb-like domains at N terminal (Figure S2). Surprisingly, the top nine blast-hits have similar amino acid sequence lengths (399–402 aa) and 73%–100% amino acid sequence identities as BZCON1 (Figure 5 and Table S1). BZCON1 and its top nine blast-hits were clustered into a group with bootstrap support of 100%, belonging to three fungal family of subclass Pleosporomycetidae (Figure S1). However, the most related to the group lost approximately 200 to 300 amino acid residues in the C-terminal region (*e.g.*, *Tuber melanosporum* and *Pyronema omphalodes* in Figure 6 and Figure S1).

## Discussion

Conidiogenesis is a widespread morphogenetic process that filamentous fungi undertake for dispersal, and also is a key step in the colonization of host plants by many fungal pathogens (Roncal *et al.* 2002; Kim *et al.* 2009). Some classical and biochemical studies have provided fundamental information on the conidiation in *C. carbonum* (Welz and Leonard 1993, 1994). Nevertheless, little is known about the molecular mechanisms controlling the development of conidia in this fungus. In the present study, for the first time, we characterized a gene *BZcon1* that is critical in controlling the conidiation of *C. carbonum*. *BZcon1* encodes a putative transcription factor (TF) that may regulate the conidiation of *C. carbonum* because we found there are two common conserved SANT/Myb-like domains in the N-terminal of BZCON1, consistent with the findings from previous studies (Grotewold *et al.* 1991; Wieser and Adams 1995; Aasland *et al.* 1996; Boyer *et al.* 2002). But we still need further functional experiments to verify this inference.

Among fungi, in addition to *BZcon1* of *C. carbonum*, only a Sant/Myb DNA-binding domain–related gene *FlbD* is known to control conidiophore development in *A. nidulans* (Wieser and Adams 1995), but there is no identifiable role for conidiation in the other Sant/Myb-like genes (*rca-1* of *Neurospora crassa* and *MYT2* of *Gibberella zeae*) (Shen *et al.* 1998; Lin *et al.* 2012). *MYT2* might have an important regulatory role for the genes required for cell proliferation and differentiation during perithecium development in *G. zeae*. *FlbD* is required for peridium formation during sexual development but not for ascospore production. In contrast, *BZcon1* mutations have no effect on the sexual growth of *C. carbonum* (Figure 3).

To date, studies of the molecular mechanism of fungal conidiation focused primarily on several model organisms, including the unicellular yeast, *Saccharomyces cerevisiae* (Sheu *et al.* 2000), filamentous saprophytic fungi (*Aspergillus nidulans* and *Neurospora crassa*) (Park and Yu 2012), human fungal pathogens (*Candida albicans* and *Cryptococcus neoformans*) (Saporito-Irwin *et al.* 1995; Bahn and Sundstrom 2001; Vallim *et al.* 2004), and plant fungal pathogens (*Ustilago maydis* and *Magnaporthe grisea*) (Kruger *et al.* 2000; Durrenberger *et al.* 2001; Yi *et al.* 2008). Interestingly, we also found the presence of *BZcon1* homologs in *Aspergillus nidulans*, *Neurospora crassa*, and *Candida albicans* (Table S1 and Figure S1). Thus, these findings suggest that *C. carbonum* should also be a candidate model fungus with great potential to provide new insights into the molecular mechanisms in the conidiation of fungi.

Our results showed that *BZcon1* was directly involved in activating the development of conidiophores, because the *BZcon1* mutants lost the conidia production capacity. In addition, our findings also indicated that the presence or absence of 126 continuous nucleotides in upstream putative promoter region of the gene *BZcon1* may potentially mediate *BZcon1* transcription. However, we also noted that the strain S129 can produce tiny numbers of conidia on PSA (potato sugar agar) but not on PDA. Yet, we found that *BZcon1* was not expressed in the sexual reproduction of the isolate S129. Meanwhile, M113-1 (*BZcon1* mutants of Aa113) could not produce any conidia on PSA. Thus, it is conceivable that although *BZcon1* is essential for the conidiation in *C. carbonum*, other gene(s) might be involved. Future efforts need to be strengthened for discovering more functional genes modulating the conidiation in *C. carbonum*.

## Supplementary Material

Supporting Information
