# Supplementary material for: BZcon1, a SANT/Myb-Type Gene Involved in the Conidiation of Cochliobolus carbonum
Source: G3 (Bethesda). 2014 Jun 3;4(8):1445–53. doi: 10.1534/g3.114.012286 (PMC4132175; doi:10.1534/g3.114.012286)
Supplement: Supporting Information [file supp_4_8_1445__index.html]

BZcon1, a SANT/Myb-Type Gene Involved in the Conidiation of Cochliobolus carbonum — Supporting Information 

# *BZcon1*, a SANT/Myb-Type Gene Involved in the Conidiation of *Cochliobolus carbonum*

## Supporting Information for Zhang *et al.*, 2014

**Files in this Data Supplement:**

- Supporting Information - Figures S1-S2 and Table S1 (PDF, 2 MB)
- Figure S1 - Phylogenetic relationships across *BZcon1* and its 164 homologs based on amino acid sequence alignment. (PDF, 1 MB)
- Figure S2 - Amino acid variations in the N-terminal regions with two SANT/Myb-like domains across *BZcon1* and its 164 homologs. (PDF, 1 MB)
- Table S1 - Summary of 164 homologs for *BZcon1*. (PDF, 144 KB)
