# Supplementary material for: BZcon1, a SANT/Myb-Type Gene Involved in the Conidiation of Cochliobolus carbonum
Source: G3 (Bethesda). 2014 Jun 3;4(8):1445–53. doi: 10.1534/g3.114.012286 (PMC4132175; doi:10.1534/g3.114.012286)
Supplement: Supporting Information [file supp_g3.114.012286_012286SI.pdf]

G3·Genes | Genomes | Genetics

BZcon1, a SANT/Myb-type gene involved in the conidiation of *Cochliobolus carbonum*

Jun-xiang Zhang,* Yi-xin Wu,§ Honhing Ho,† Hao Zhang,§ Peng-fei He,§ Yue-qiu He*

*Faculty of Plant Protection, Yunnan Agricultural University, Kunming 650201, Yunnan, China
§Faculty of Agriculture and Biotechnology, Yunnan Agricultural University, Kunming 650201, Yunnan, China
†Department of Biology, State University of New York, New Paltz, NY 12561, USA

Correspondence to
Yue-qiu He
Faculty of Plant Protection, Yunnan Agricultural University, Kunming 650201, Yunnan, China
Phone: 86-0871-65228221
Fax: 86-0871-65228532
Email: ynfh2007@163.com

DOI: 10.1534/g3.114.012286

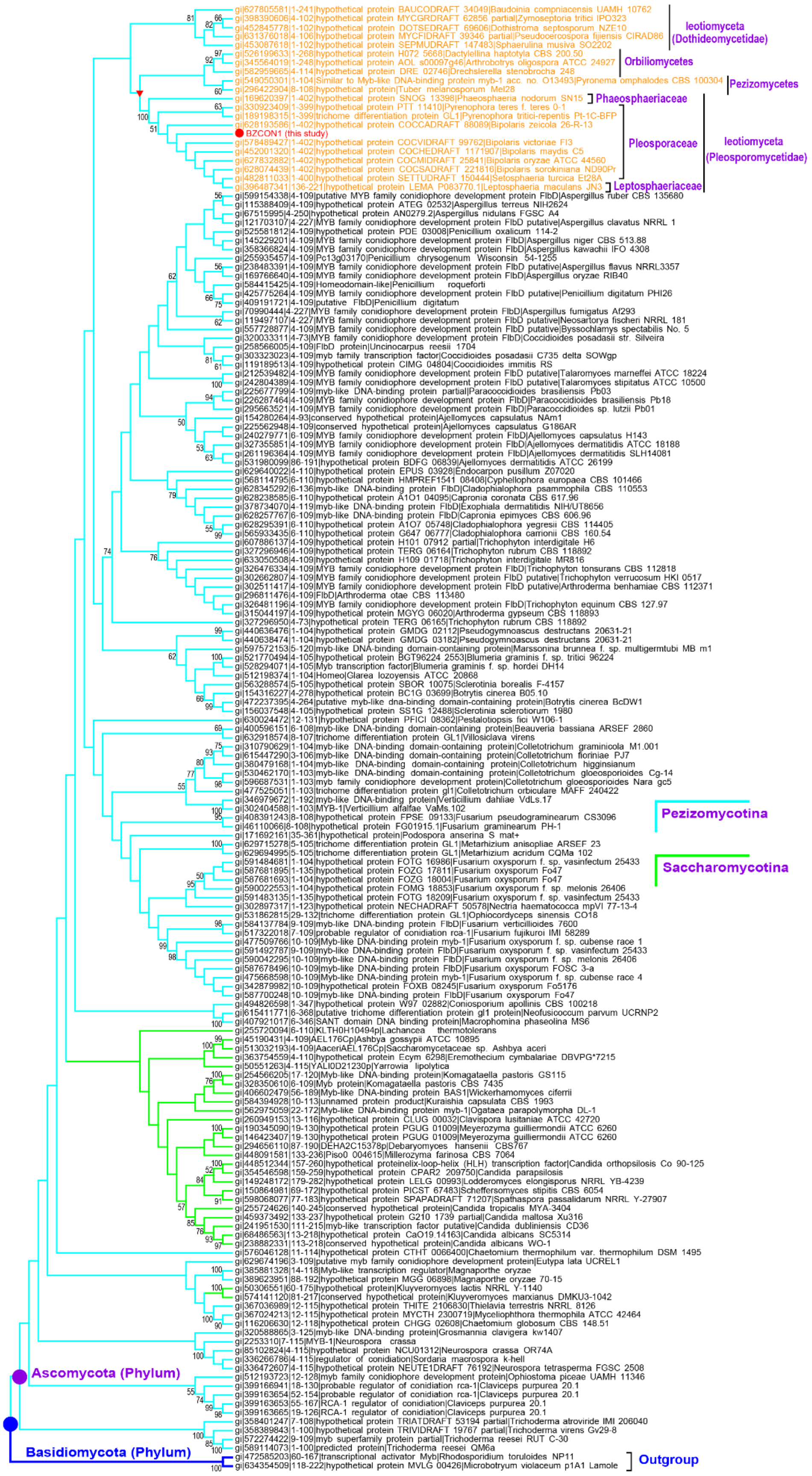

Figure S1 Phylogenetic relationships across *BZcon1* and its 164 homologs based on amino acid sequence alignment. The bootstrap support values higher than 50% were shown upon the tree.

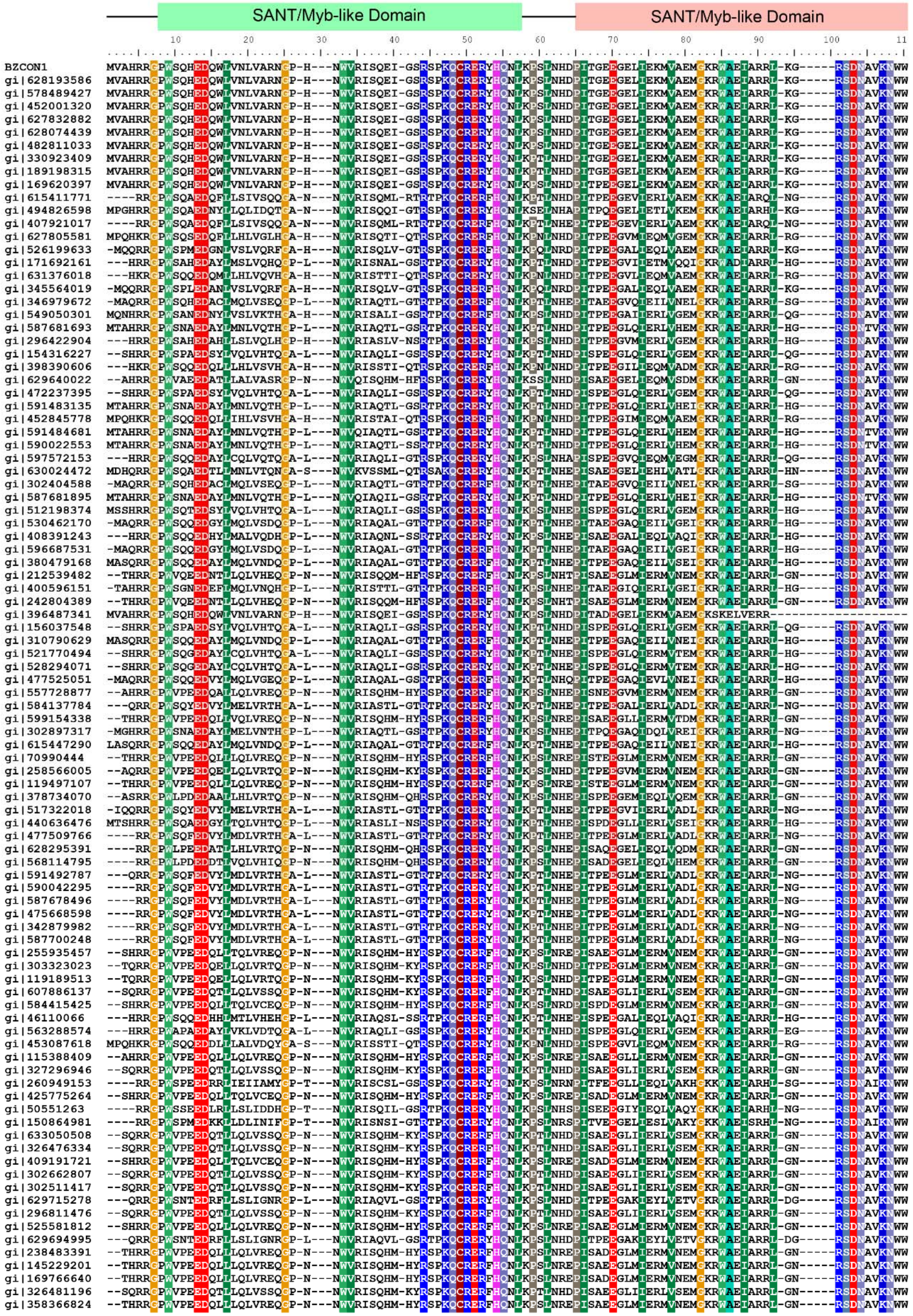

Figure S2 Amino acid variations in the N-terminal regions with two SANT/Myb-like domains across *BZcon1* and its 164 homologs. Two SANT/Myb-like domains were shown by boxes. Conserved amino acid residues shared by 95% of *BZcon1* and its 164 homologs were marked by colors.

Table S1 Summary of 164 homologs for *BZcon1*.

GI	Alignment Position	Coverage	E value	Identity	Function Annotations	Origin
628193586	1-402	100%	0	100%	hypothetical_protein_COCCADRAFT_88089	Bipolaris_zeicola_26-R-13
452001320	1-402	100%	0	99%	hypothetical_protein_COCHEDRAFT_1171907	Bipolaris_maydis_C5
627832882	1-402	100%	0	99%	hypothetical_protein_COCMIDRAFT_25841	Bipolaris_oryzae_ATCC_44560
628074439	1-402	100%	0	99%	hypothetical_protein_COCSADRAFT_221816	Bipolaris_sorokiniana_ND90Pr
578489427	1-402	100%	0	99%	hypothetical_protein_COCVIDRAFT_99762	Bipolaris_victoriae_FI3
482811033	1-400	100%	0	92%	hypothetical_protein_SETTUDRAFT_150444	Setosphaeria_turcica_Et28A
330923409	1-399	100%	0	84%	hypothetical_protein_PTT_11410	Pyrenophora_teres_f._teres_0-1
189198315	1-399	100%	0	83%	trichome_differentiation_protein_GL1	Pyrenophora_tritici-repentis_Pt-1C-BFP
169620397	1-402	100%	0	73%	hypothetical_protein_SNOG_13398	Phaeosphaeria_nodorum_SN15
615411771	6-368	94%	8.00E-77	45%	putative_trichome_differentiation_protein_gl1_protein	Neofusicoccum_parvum_UCRNP2
494826598	1-347	89%	6.00E-76	47%	hypothetical_protein_W97_02882	Coniosporium_apollinis_CBS_100218
407921017	6-346	86%	3.00E-72	45%	SANT_domain_DNA_binding_protein	Macrophomina_phaseolina_MS6
627805581	1-241	65%	5.00E-57	50%	hypothetical_protein_BAUCODRAFT_34049	Baudoinia_compniacensis_UAMH_10762
526199633	1-268	76%	4.00E-53	45%	hypothetical_protein_H072_5668	Dactylellina_haptotyla_CBS_200.50
171692161	35-361	87%	2.00E-52	40%	hypothetical_protein	Podospora_anserina_S_mat+
631376018	4-106	25%	6.00E-52	82%	hypothetical_protein_MYCFIDRAFT_39346_partial	Pseudocercospora_fijiensis_CIRAD86
345564019	1-248	69%	2.00E-51	47%	hypothetical_protein_AOL_s00097g46	Arthrobotrys_oligospora_ATCC_24927
346979672	1-192	46%	4.00E-50	51%	myb-like_DNA-binding_protein	Verticillium_dahliae_VdLs.17
549050301	1-104	25%	4.00E-50	78%	Similar_to_Myb-like_DNA-binding_protein_myb-1_acc._no._O13493	Pyronema_omphalodes_CBS_100304
587681693	1-104	25%	2.00E-49	77%	hypothetical_protein_FOZG_18004	Fusarium_oxysporum_Fo47
154316227	4-278	80%	4.00E-49	42%	hypothetical_protein_BC1G_03699	Botrytis_cinerea_B05.10
296422904	8-108	25%	4.00E-49	78%	hypothetical_protein	Tuber_melanosporum_Mel28
398390606	4-102	24%	5.00E-49	80%	hypothetical_protein_MYCGRDRAFT_62856_partial	Zymoseptoria_tritici_IPO323
629640022	4-110	26%	6.00E-49	77%	hypothetical_protein_EPUS_03928	Endocarpon_pusillum_Z07020
472237395	4-264	76%	7.00E-49	42%	putative_myb-like_dna-binding_domain-containing_protein	Botrytis_cinerea_BcDW1
591483135	1-135	33%	8.00E-49	65%	hypothetical_protein_FOTG_18209	Fusarium_oxysporum_f._sp._vasinfectum_25433
452845778	1-102	25%	9.00E-49	78%	hypothetical_protein_DOTSEDRAFT_69606	Dothistroma_septosporum_NZE10
591484681	1-104	25%	1.00E-48	76%	hypothetical_protein_FOTG_16986	Fusarium_oxysporum_f._sp._vasinfectum_25433
590022553	1-104	25%	2.00E-48	76%	hypothetical_protein_FOMG_18853	Fusarium_oxysporum_f._sp._melonis_26406
597572153	5-120	29%	5.00E-48	71%	myb-like_DNA-binding_domain-containing_protein	Marssonina_brunnea_f._sp._multigermtubi_MB_m1
587700248	10-109	33%	8.00E-48	65%	myb-like_DNA-binding_protein_FlbD	Fusarium_oxysporum_Fo47
630024472	12-131	29%	8.00E-48	67%	hypothetical_protein_PFICI_08362	Pestalotiopsis_fici_W106-1

302404588	1-103	25%	8.00E-48	74%	MYB-1	Verticillium_alfalfae_VaMs.102
530462170	1-103	25%	1.00E-47	73%	myb-like_DNA-binding_domain-containing_protein	Colletotrichum_gloeosporioides_Cg-14
512198374	1-104	25%	1.00E-47	77%	Homeo	Glarea_lozoyensis_ATCC_20868
596687531	1-103	25%	2.00E-47	73%	myb_family_conidiophore_development_protein	Colletotrichum_gloeosporioides_Nara_gc5
408391243	8-108	25%	2.00E-47	76%	hypothetical_protein_FPSE_09133	Fusarium_pseudograminearum_CS3096
380479168	1-104	25%	5.00E-47	72%	myb-like_DNA-binding_domain-containing_protein	Colletotrichum_higginsianum
212539482	4-109	26%	7.00E-47	73%	MYB_family_conidiophore_development_protein_FlbD_putative	Talaromyces_marneffei_ATCC_18224
400596151	6-108	25%	7.00E-47	74%	myb-like_DNA-binding_domain-containing_protein	Beauveria_bassiana_ARSEF_2860
242804389	4-109	26%	1.00E-46	73%	MYB_family_conidiophore_development_protein_FlbD_putative	Talaromyces_stipitatus_ATCC_10500
156037548	4-105	25%	1.00E-46	76%	hypothetical_protein_SS1G_12488	Sclerotinia_sclerotiorum_1980
396487341	136-221	21%	1.00E-46	91%	hypothetical_protein_LEMA_P083770.1	Leptosphaeria_maculans_JN3
528294071	4-105	25%	2.00E-46	76%	Myb_transcription_factor	Blumeria_graminis_f._sp._hordei_DH14
521770494	4-105	25%	2.00E-46	76%	hypothetical_protein_BGT96224_2553	Blumeria_graminis_f._sp._tritici_96224
310790629	1-104	25%	2.00E-46	72%	myb-like_DNA-binding_domain-containing_protein	Colletotrichum_graminicola_M1.001
477525051	1-103	25%	2.00E-46	74%	trichome_differentiation_protein_gl1	Colletotrichum_orbiculare_MAFF_240422
557728877	4-109	26%	3.00E-46	74%	MYB_family_conidiophore_development_protein_FlbD_putative	Byssochlamys_spectabilis_No._5
302897317	1-123	30%	4.00E-46	68%	hypothetical_protein_NECHADRAFT_50578	Nectria_haematococca_mpVI_77-13-4
599154338	4-109	26%	4.00E-46	73%	putative_MYB_family_conidiophore_development_protein_FlbD	Aspergillus_ruber_CBS_135680
615447290	3-106	25%	4.00E-46	71%	myb-like_DNA-binding_domain-containing_protein	Colletotrichum_fioriniae_PJ7
584137784	9-109	25%	4.00E-46	73%	myb-like_DNA-binding_protein_FlbD	Fusarium_verticillioides_7600
70990444	4-227	59%	5.00E-46	45%	MYB_family_conidiophore_development_protein_FlbD	Aspergillus_fumigatus_Af293
258566005	4-109	26%	5.00E-46	75%	FlbD_protein	Uncinocarpus_reesii_1704
119497107	4-227	59%	6.00E-46	44%	MYB_family_conidiophore_development_protein_FlbD_putative	Neosartorya_fischeri_NRRL_181
378734070	4-119	28%	6.00E-46	71%	myb-like_DNA-binding_protein_FlbD	Exophiala_dermatitidis_NIH/UT8656
517322018	7-109	25%	6.00E-46	71%	probable_regulator_of_conidiation_rca-1	Fusarium_fujikuroi_IMI_58289
440636476	1-104	25%	6.00E-46	73%	hypothetical_protein_GMDG_02112	Pseudogymnoascus_destructans_20631-21
477509766	10-109	24%	7.00E-46	73%	Myb-like_DNA-binding_protein_myb-1	Fusarium_oxysporum_f._sp._cubense_race_1
628295391	6-110	26%	8.00E-46	73%	hypothetical_protein_A1O7_05748	Cladophialophora_yegresii_CBS_114405
303323023	4-109	26%	1.00E-45	75%	myb_family_transcription_factor	Coccidioides_posadasii_C735_delta_SOWgp
568114795	6-110	26%	1.00E-45	75%	hypothetical_protein_HMPREF1541_08408	Cyphellophora_europaea_CBS_101466
255935457	4-109	26%	1.00E-45	73%	Pc13g03170	Penicillium_chrysogenum_Wisconsin_54-1255
591492787	9-109	25%	1.00E-45	72%	myb-like_DNA-binding_protein_FlbD	Fusarium_oxysporum_f._sp._vasinfectum_25433
475668598	10-109	24%	1.00E-45	73%	Myb-like_DNA-binding_protein_myb-1	Fusarium_oxysporum_f._sp._cubense_race_4
590042295	10-109	24%	1.00E-45	73%	myb-like_DNA-binding_protein_FlbD	Fusarium_oxysporum_f._sp._melonis_26406
587681895	1-135	24%	1.00E-45	73%	hypothetical_protein_FOZG_17811	Fusarium_oxysporum_Fo47

342879982	10-109	24%	1.00E-45	73%	hypothetical_protein_FOXB_08245	Fusarium_oxysporum_Fo5176
587678496	10-109	24%	1.00E-45	73%	myb-like_DNA-binding_protein_FlbD	Fusarium_oxysporum_FOSC_3-a
115388409	4-109	26%	2.00E-45	74%	hypothetical_protein_ATEG_02532	Aspergillus_terreus_NIH2624
119189513	4-109	26%	2.00E-45	75%	hypothetical_protein_CIMG_04804	Coccidioides_immitis_RS
584415425	4-109	26%	2.00E-45	73%	Homeodomain-like	Penicillium_roqueforti
607886137	4-109	26%	2.00E-45	71%	hypothetical_protein_H101_07912_partial	Trichophyton_interdigitale_H6
46110066	8-108	25%	2.00E-45	74%	hypothetical_protein_FG01915.1	Fusarium_graminearum_PH-1
563288574	5-105	25%	2.00E-45	76%	hypothetical_protein_SBOR_10075	Sclerotinia_borealis_F-4157
453087618	1-102	25%	2.00E-45	76%	hypothetical_protein_SEPMUDRAFT_147483	Sphaerulina_musiva_SO2202
50551263	4-115	27%	3.00E-45	71%	YALI0D21230p	Yarrowia_lipolytica
409191721	4-109	26%	3.00E-45	72%	putative_FlbD	Penicillium_digitatum
425775264	4-109	26%	3.00E-45	72%	MYB_family_conidiophore_development_protein_FlbD_putative	Penicillium_digitatum_PHI26
633050508	4-109	26%	3.00E-45	71%	hypothetical_protein_H109_01718	Trichophyton_interdigitale_MR816
327296946	4-109	26%	3.00E-45	71%	hypothetical_protein_TERG_06164	Trichophyton_rubrum_CBS_118892
326476334	4-109	26%	3.00E-45	71%	MYB_family_conidiophore_development_protein_FlbD	Trichophyton_tonsurans_CBS_112818
260949153	13-116	25%	3.00E-45	73%	hypothetical_protein_CLUG_00032	Clavispora_lusitaniae_ATCC_42720
150864981	69-172	25%	3.00E-45	72%	hypothetical_protein_PICST_67483	Scheffersomyces_stipitis_CBS_6054
302511417	4-109	26%	4.00E-45	71%	MYB_family_conidiophore_development_protein_FlbD_putative	Arthroderma_benhamiae_CBS_112371
302662807	4-109	26%	4.00E-45	71%	MYB_family_conidiophore_development_protein_FlbD_putative	Trichophyton_verrucosum_HKI_0517
296811476	4-109	26%	5.00E-45	71%	FlbD	Arthroderma_otae_CBS_113480
525581812	4-109	26%	5.00E-45	72%	hypothetical_protein_PDE_03008	Penicillium_oxalicum_114-2
629715278	5-105	25%	5.00E-45	74%	trichome_differentiation_protein_GL1	Metarhizium_anisopliae_ARSEF_23
629694995	5-105	25%	7.00E-45	74%	trichome_differentiation_protein_GL1	Metarhizium_acridum_CQMa_102
238483391	4-109	26%	1.00E-44	71%	MYB_family_conidiophore_development_protein_FlbD_putative	Aspergillus_flavus_NRRL3357
358366824	4-109	26%	1.00E-44	72%	MYB_family_conidiophore_development_protein_FlbD	Aspergillus_kawachii_IFO_4308
145229201	4-109	26%	1.00E-44	72%	MYB_family_conidiophore_development_protein_FlbD	Aspergillus_niger_CBS_513.88
169766640	4-109	26%	1.00E-44	71%	MYB_family_conidiophore_development_protein_FlbD	Aspergillus_oryzae_RIB40
326481196	4-109	26%	1.00E-44	71%	MYB_family_conidiophore_development_protein_FlbD	Trichophyton_equinum_CBS_127.97
315044197	4-109	26%	2.00E-44	71%	hypothetical_protein_MGYG_06020	Arthroderma_gypseum_CBS_118893
565933435	6-110	26%	3.00E-44	73%	hypothetical_protein_G647_06777	Cladophialophora_carrionii_CBS_160.54
531862815	29-132	25%	3.00E-44	71%	trichome_differentiation_protein_GL1	Ophiocordyceps_sinensis_CO18
632918574	8-107	24%	3.00E-44	74%	trichome_differentiation_protein_GL1	Villosiclava_virens
628238585	6-110	26%	4.00E-44	73%	hypothetical_protein_A1O1_04095	Capronia_coronata_CBS_617.96
295663521	4-109	26%	4.00E-44	72%	MYB_family_conidiophore_development_protein_FlbD	Paracoccidioides_sp._lutzii_Pb01
628345292	6-136	32%	7.00E-44	64%	myb-like_DNA-binding_protein_FlbD	Cladophialophora_psammophila_CBS_110553

225562948	4-109	26%	7.00E-44	72%	conserved_hypothetical_protein	Ajellomyces_capsulatus_G186AR
628257767	6-109	25%	7.00E-44	73%	myb-like_DNA-binding_protein_FlbD	Capronia_epimyces_CBS_606.96
225677799	4-109	26%	9.00E-44	72%	myb-like_DNA-binding_protein_partial	Paracoccidioides_brasiliensis_Pb03
327355851	4-109	26%	1.00E-43	72%	MYB_family_conidiophore_development_protein_FlbD	Ajellomyces_dermatitidis_ATCC_18188
261196364	4-109	26%	1.00E-43	72%	MYB_family_conidiophore_development_protein_FlbD	Ajellomyces_dermatitidis_SLH14081
226287464	4-109	26%	1.00E-43	72%	MYB_family_conidiophore_development_protein_FlbD	Paracoccidioides_brasiliensis_Pb18
440638474	1-104	25%	1.00E-43	70%	hypothetical_protein_GMDG_03182	Pseudogymnoascus_destructans_20631-21
582959665	4-114	27%	2.00E-43	70%	hypothetical_protein_DRE_02746	Drechslerella_stenobrocha_248
240279771	6-109	25%	2.00E-43	72%	MYB_family_conidiophore_development_protein_FlbD	Ajellomyces_capsulatus_H143
328350610	6-109	25%	2.00E-43	70%	Myb_protein	Komagataella_pastoris_CBS_7435
254566205	17-120	25%	2.00E-43	70%	Myb-like_DNA-binding_protein	Komagataella_pastoris_GS115
531980099	86-191	26%	3.00E-43	72%	hypothetical_protein_BDFG_06839	Ajellomyces_dermatitidis_ATCC_26199
190345090	19-130	27%	4.00E-43	67%	hypothetical_protein_PGUG_01009	Meyerozyma_guilliermondii_ATCC_6260
146423407	19-130	27%	5.00E-43	67%	hypothetical_protein_PGUG_01009	Meyerozyma_guilliermondii_ATCC_6260
598068077	77-183	26%	5.00E-43	69%	hypothetical_protein_SPAPADRAFT_71207	Spathaspora_passalidarum_NRRL_Y-27907
149248172	179-282	25%	8.00E-43	70%	hypothetical_protein_LELG_00993	Lodderomyces_elongisporus_NRRL_YB-4239
406602479	56-189	33%	1.00E-42	60%	Myb-like_DNA-binding_protein_BAS1	Wickerhamomyces_ciferrii
448091581	133-236	25%	1.00E-42	70%	Piso0_004615	Millerozyma_farinosa_CBS_7064
67515995	4-250	62%	7.00E-42	43%	hypothetical_protein_AN0279.2	Aspergillus_nidulans_FGSC_A4
448512344	157-260	25%	9.00E-42	68%	hypothetical_proteinelix-loop-helix_(HLH)_transcription_factor	Candida_orthopsilosis_Co_90-125
45190431	4-109	26%	3.00E-41	64%	AEL176Cp	Ashbya_gossypii_ATCC_10895
513032193	4-109	26%	3.00E-41	64%	AaceriAEL176Cp	Saccharomycetaceae_sp._Ashbya_aceri
363754559	4-110	26%	4.00E-41	63%	hypothetical_protein_Ecym_6298	Eremothecium_cymbalariae_DBVPG*7215
241951530	111-215	26%	1.00E-40	65%	myb-like_transcription_factor_putative	Candida_dubliniensis_CD36
629674196	3-109	26%	1.00E-40	62%	putative_myb_family_conidiophore_development_protein	Eutypa_lata_UCREL1
584394928	10-113	25%	2.00E-40	67%	unnamed_protein_product	Kuraishia_capsulata_CBS_1993
459373492	133-237	26%	4.00E-40	67%	hypothetical_protein_G210_1739_partial	Candida_maltosa_Xu316
294656110	87-190	25%	4.00E-40	66%	DEHA2C15378p	Debaryomyces_hansenii_CBS767
68486563	113-218	26%	8.00E-40	64%	hypothetical_protein_CaO19.14163	Candida_albicans_SC5314
399166941	18-130	28%	9.00E-40	65%	probable_regulator_of_conidiation_rca-1	Claviceps_purpurea_20.1
238882331	113-218	26%	9.00E-40	64%	conserved_hypothetical_protein	Candida_albicans_WO-1
354546598	159-259	25%	1.00E-39	68%	hypothetical_protein_CPAR2_209750	Candida_parapsilosis
255724626	140-245	26%	4.00E-39	65%	conserved_hypothetical_protein	Candida_tropicalis_MYA-3404
320588865	3-125	31%	5.00E-39	59%	myb-like_DNA-binding_protein	Grosmannia_clavigera_kw1407
255720094	6-110	25%	1.00E-38	64%	KLTH0H10494p	Lachancea_thermotolerans

562975059	22-172	33%	3.00E-37	53%	Myb-like_DNA-binding_protein_myb-1	Ogataea_parapolymorpha_DL-1
85102824	4-115	27%	3.00E-36	60%	hypothetical_protein_NCU01312	Neurospora_crassa_OR74A
2253310	7-115	27%	4.00E-36	59%	MYB-1	Neurospora_crassa
336266786	4-115	27%	5.00E-36	59%	regulator_of_conidiation	Sordaria_macrospora_k-hell
336472607	4-115	27%	8.00E-36	59%	hypothetical_protein_NEUTE1DRAFT_76192	Neurospora_tetrasperma_FGSC_2508
121703107	4-227	61%	1.00E-35	41%	MYB_family_conidiophore_development_protein_FlbD_putative	Aspergillus_clavatus_NRRL_1
576046128	11-114	25%	1.00E-34	62%	hypothetical_protein_CTHT_0066400	Chaetomium_thermophilum_var._thermophilum_DSM_1495
154280264	4-93	26%	1.00E-32	61%	conserved_hypothetical_protein	Ajellomyces_capsulatus_NAm1
320033311	4-73	17%	1.00E-31	84%	MYB_family_conidiophore_development_protein_FlbD	Coccidioides_posadasii_str._Silveira
327296950	4-73	17%	3.00E-31	79%	hypothetical_protein_TERG_06165	Trichophyton_rubrum_CBS_118892
399163653	55-167	28%	8.00E-30	54%	RCA-1_regulator_of_conidiation	Claviceps_purpurea_20.1
385881328	14-118	25%	2.00E-29	56%	Myb-like_transcription_regulator	Magnaporthe_oryzae
389623951	88-192	25%	2.00E-29	56%	hypothetical_protein_MGG_06898	Magnaporthe_oryzae_70-15
399163665	19-126	26%	2.00E-28	52%	RCA-1_regulator_of_conidiation	Claviceps_purpurea_20.1
50306551	60-175	28%	1.00E-27	48%	hypothetical_protein	Kluyveromyces_lactis_NRRL_Y-1140
399163654	52-154	25%	1.00E-27	50%	probable_regulator_of_conidiation_rca-1	Claviceps_purpurea_20.1
358401247	7-108	25%	4.00E-27	54%	hypothetical_protein_TRIATDRAFT_53194_partial	Trichoderma_atroviride_IMI_206040
574141120	81-217	34%	5.00E-27	43%	conserved_hypothetical_protein	Kluyveromyces_marxianus_DMKU3-1042
472585203	60-167	26%	3.00E-26	52%	transcriptional_activator_Myb	Rhodosporidium_toruloides_NP11
367024213	12-115	26%	8.00E-26	55%	hypothetical_protein_MYCTH_2300719	Myceliophthora_thermophila_ATCC_42464
572274422	9-109	25%	1.00E-25	52%	myb_superfamily_protein_partial	Trichoderma_reesei_RUT_C-30
589114073	1-100	24%	2.00E-25	53%	predicted_protein	Trichoderma_reesei_QM6a
358389843	1-100	24%	2.00E-25	54%	hypothetical_protein_TRIVIDRAFT_19767_partial	Trichoderma_virens_Gv29-8
116206630	12-118	26%	1.00E-24	51%	hypothetical_protein_CHGG_02608	Chaetomium_globosum_CBS_148.51
512193723	12-128	26%	2.00E-24	50%	myb_family_conidiophore_development_protein	Ophiostoma_piceae_UAMH_11346
367036989	12-115	26%	2.00E-24	54%	hypothetical_protein_THITE_2106830	Thielavia_terrestris_NRRL_8126
634354509	118-222	25%	2.00E-24	52%	hypothetical_protein_MVLG_00426	Microbotryum_violaceum_p1A1_Lamole
