# Supplementary material for: BZcon1, a SANT/Myb-Type Gene Involved in the Conidiation of Cochliobolus carbonum
Source: G3 (Bethesda). 2014 Jun 3;4(8):1445–53. doi: 10.1534/g3.114.012286 (PMC4132175; doi:10.1534/g3.114.012286)
Supplement: Supporting Information [file supp_g3.114.012286_FigureS1.pdf]

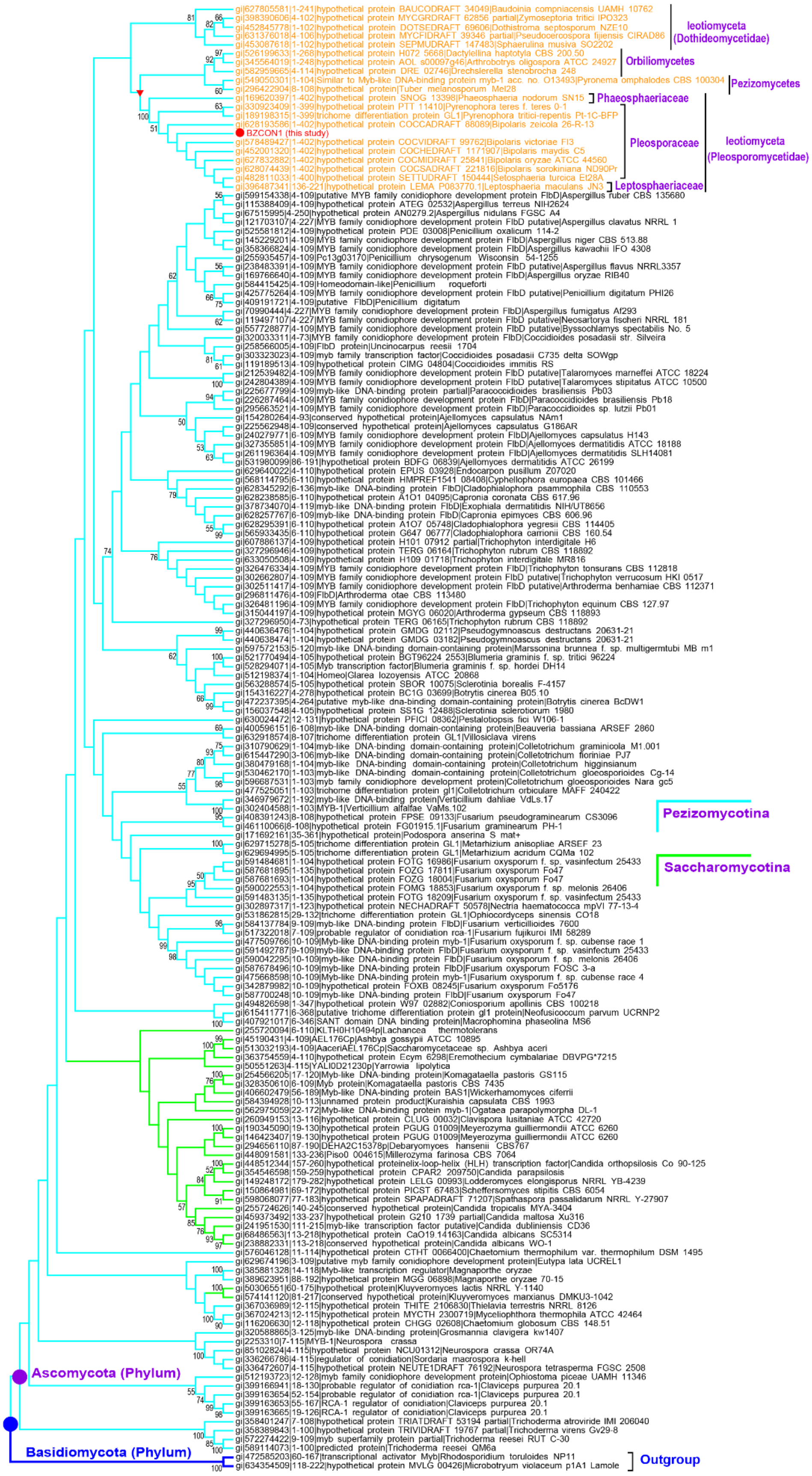

Figure S1 Phylogenetic relationships across *BZcon1* and its 164 homologs based on amino acid sequence alignment. The bootstrap support values higher than 50% were shown upon the tree.
