# Supplementary material for: BZcon1, a SANT/Myb-Type Gene Involved in the Conidiation of Cochliobolus carbonum
Source: G3 (Bethesda). 2014 Jun 3;4(8):1445–53. doi: 10.1534/g3.114.012286 (PMC4132175; doi:10.1534/g3.114.012286)
Supplement: Supporting Information [file supp_g3.114.012286_FigureS2.pdf]

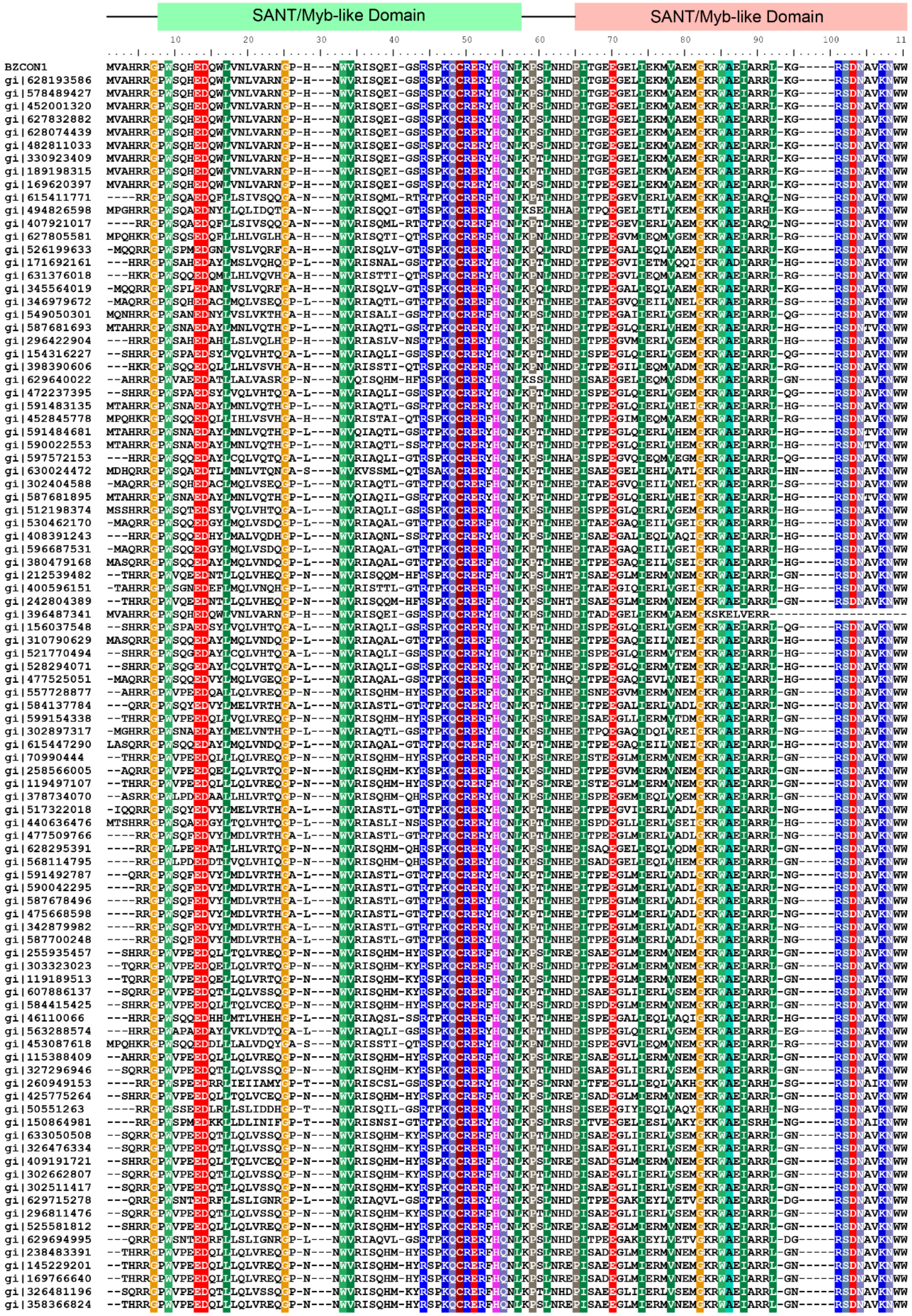

Figure S2 Amino acid variations in the N-terminal regions with two SANT/Myb-like domains across *BZcon1* and its 164 homologs. Two SANT/Myb-like domains were shown by boxes. Conserved amino acid residues shared by 95% of *BZcon1* and its 164 homologs were marked by colors.
