# Supplementary material for: BZcon1, a SANT/Myb-Type Gene Involved in the Conidiation of Cochliobolus carbonum
Source: G3 (Bethesda). 2014 Jun 3;4(8):1445–53. doi: 10.1534/g3.114.012286 (PMC4132175; doi:10.1534/g3.114.012286)
Supplement: Supporting Information [file supp_g3.114.012286_TableS1.pdf]

Table S1 Summary of 164 homologs for *BZcon1*.

GI	Alignment Position	Coverage	E value	Identity	Function Annotations	Origin
628193586	1-402	100%	0	100%	hypothetical_protein_COCCADRAFT_88089	Bipolaris_zeicola_26-R-13
452001320	1-402	100%	0	99%	hypothetical_protein_COCHEDRAFT_1171907	Bipolaris_maydis_C5
627832882	1-402	100%	0	99%	hypothetical_protein_COCMIDRAFT_25841	Bipolaris_oryzae_ATCC_44560
628074439	1-402	100%	0	99%	hypothetical_protein_COCSADRAFT_221816	Bipolaris_sorokiniana_ND90Pr
578489427	1-402	100%	0	99%	hypothetical_protein_COCVIDRAFT_99762	Bipolaris_victoriae_FI3
482811033	1-400	100%	0	92%	hypothetical_protein_SETTUDRAFT_150444	Setosphaeria_turcica_Et28A
330923409	1-399	100%	0	84%	hypothetical_protein_PTT_11410	Pyrenophora_teres_f._teres_0-1
189198315	1-399	100%	0	83%	trichome_differentiation_protein_GL1	Pyrenophora_tritici-repentis_Pt-1C-BFP
169620397	1-402	100%	0	73%	hypothetical_protein_SNOG_13398	Phaeosphaeria_nodorum_SN15
615411771	6-368	94%	8.00E-77	45%	putative_trichome_differentiation_protein_gl1_protein	Neofusicoccum_parvum_UCRNP2
494826598	1-347	89%	6.00E-76	47%	hypothetical_protein_W97_02882	Coniosporium_apollinis_CBS_100218
407921017	6-346	86%	3.00E-72	45%	SANT_domain_DNA_binding_protein	Macrophomina_phaseolina_MS6
627805581	1-241	65%	5.00E-57	50%	hypothetical_protein_BAUCODRAFT_34049	Baudoinia_compniacensis_UAMH_10762
526199633	1-268	76%	4.00E-53	45%	hypothetical_protein_H072_5668	Dactylellina_haptotyla_CBS_200.50
171692161	35-361	87%	2.00E-52	40%	hypothetical_protein	Podospora_anserina_S_mat+
631376018	4-106	25%	6.00E-52	82%	hypothetical_protein_MYCFIDRAFT_39346_partial	Pseudocercospora_fijiensis_CIRAD86
345564019	1-248	69%	2.00E-51	47%	hypothetical_protein_AOL_s00097g46	Arthrobotrys_oligospora_ATCC_24927
346979672	1-192	46%	4.00E-50	51%	myb-like_DNA-binding_protein	Verticillium_dahliae_VdLs.17
549050301	1-104	25%	4.00E-50	78%	Similar_to_Myb-like_DNA-binding_protein_myb-1_acc._no._O13493	Pyronema_omphalodes_CBS_100304
587681693	1-104	25%	2.00E-49	77%	hypothetical_protein_FOZG_18004	Fusarium_oxysporum_Fo47
154316227	4-278	80%	4.00E-49	42%	hypothetical_protein_BC1G_03699	Botrytis_cinerea_B05.10
296422904	8-108	25%	4.00E-49	78%	hypothetical_protein	Tuber_melanosporum_Mel28
398390606	4-102	24%	5.00E-49	80%	hypothetical_protein_MYCGRDRAFT_62856_partial	Zymoseptoria_tritici_IPO323
629640022	4-110	26%	6.00E-49	77%	hypothetical_protein_EPUS_03928	Endocarpon_pusillum_Z07020
472237395	4-264	76%	7.00E-49	42%	putative_myb-like_dna-binding_domain-containing_protein	Botrytis_cinerea_BcDW1
591483135	1-135	33%	8.00E-49	65%	hypothetical_protein_FOTG_18209	Fusarium_oxysporum_f._sp._vasinfectum_25433
452845778	1-102	25%	9.00E-49	78%	hypothetical_protein_DOTSEDRAFT_69606	Dothistroma_septosporum_NZE10
591484681	1-104	25%	1.00E-48	76%	hypothetical_protein_FOTG_16986	Fusarium_oxysporum_f._sp._vasinfectum_25433
590022553	1-104	25%	2.00E-48	76%	hypothetical_protein_FOMG_18853	Fusarium_oxysporum_f._sp._melonis_26406
597572153	5-120	29%	5.00E-48	71%	myb-like_DNA-binding_domain-containing_protein	Marssonina_brunnea_f._sp._multigermtubi_MB_m1
587700248	10-109	33%	8.00E-48	65%	myb-like_DNA-binding_protein_FlbD	Fusarium_oxysporum_Fo47
630024472	12-131	29%	8.00E-48	67%	hypothetical_protein_PFICI_08362	Pestalotiopsis_fici_W106-1

302404588	1-103	25%	8.00E-48	74%	MYB-1	Verticillium_alfalfae_VaMs.102
530462170	1-103	25%	1.00E-47	73%	myb-like_DNA-binding_domain-containing_protein	Colletotrichum_gloeosporioides_Cg-14
512198374	1-104	25%	1.00E-47	77%	Homeo	Glarea_lozoyensis_ATCC_20868
596687531	1-103	25%	2.00E-47	73%	myb_family_conidiophore_development_protein	Colletotrichum_gloeosporioides_Nara_gc5
408391243	8-108	25%	2.00E-47	76%	hypothetical_protein_FPSE_09133	Fusarium_pseudograminearum_CS3096
380479168	1-104	25%	5.00E-47	72%	myb-like_DNA-binding_domain-containing_protein	Colletotrichum_higginsianum
212539482	4-109	26%	7.00E-47	73%	MYB_family_conidiophore_development_protein_FlbD_putative	Talaromyces_marneffei_ATCC_18224
400596151	6-108	25%	7.00E-47	74%	myb-like_DNA-binding_domain-containing_protein	Beauveria_bassiana_ARSEF_2860
242804389	4-109	26%	1.00E-46	73%	MYB_family_conidiophore_development_protein_FlbD_putative	Talaromyces_stipitatus_ATCC_10500
156037548	4-105	25%	1.00E-46	76%	hypothetical_protein_SS1G_12488	Sclerotinia_sclerotiorum_1980
396487341	136-221	21%	1.00E-46	91%	hypothetical_protein_LEMA_P083770.1	Leptosphaeria_maculans_JN3
528294071	4-105	25%	2.00E-46	76%	Myb_transcription_factor	Blumeria_graminis_f._sp._hordei_DH14
521770494	4-105	25%	2.00E-46	76%	hypothetical_protein_BGT96224_2553	Blumeria_graminis_f._sp._tritici_96224
310790629	1-104	25%	2.00E-46	72%	myb-like_DNA-binding_domain-containing_protein	Colletotrichum_graminicola_M1.001
477525051	1-103	25%	2.00E-46	74%	trichome_differentiation_protein_gl1	Colletotrichum_orbiculare_MAFF_240422
557728877	4-109	26%	3.00E-46	74%	MYB_family_conidiophore_development_protein_FlbD_putative	Byssochlamys_spectabilis_No._5
302897317	1-123	30%	4.00E-46	68%	hypothetical_protein_NECHADRAFT_50578	Nectria_haematococca_mpVI_77-13-4
599154338	4-109	26%	4.00E-46	73%	putative_MYB_family_conidiophore_development_protein_FlbD	Aspergillus_ruber_CBS_135680
615447290	3-106	25%	4.00E-46	71%	myb-like_DNA-binding_domain-containing_protein	Colletotrichum_fioriniae_PJ7
584137784	9-109	25%	4.00E-46	73%	myb-like_DNA-binding_protein_FlbD	Fusarium_verticillioides_7600
70990444	4-227	59%	5.00E-46	45%	MYB_family_conidiophore_development_protein_FlbD	Aspergillus_fumigatus_Af293
258566005	4-109	26%	5.00E-46	75%	FlbD_protein	Uncinocarpus_reesii_1704
119497107	4-227	59%	6.00E-46	44%	MYB_family_conidiophore_development_protein_FlbD_putative	Neosartorya_fischeri_NRRL_181
378734070	4-119	28%	6.00E-46	71%	myb-like_DNA-binding_protein_FlbD	Exophiala_dermatitidis_NIH/UT8656
517322018	7-109	25%	6.00E-46	71%	probable_regulator_of_conidiation_rca-1	Fusarium_fujikuroi_IMI_58289
440636476	1-104	25%	6.00E-46	73%	hypothetical_protein_GMDG_02112	Pseudogymnoascus_destructans_20631-21
477509766	10-109	24%	7.00E-46	73%	Myb-like_DNA-binding_protein_myb-1	Fusarium_oxysporum_f._sp._cubense_race_1
628295391	6-110	26%	8.00E-46	73%	hypothetical_protein_A1O7_05748	Cladophialophora_yegresii_CBS_114405
303323023	4-109	26%	1.00E-45	75%	myb_family_transcription_factor	Coccidioides_posadasii_C735_delta_SOWgp
568114795	6-110	26%	1.00E-45	75%	hypothetical_protein_HMPREF1541_08408	Cyphellophora_europaea_CBS_101466
255935457	4-109	26%	1.00E-45	73%	Pc13g03170	Penicillium_chrysogenum_Wisconsin_54-1255
591492787	9-109	25%	1.00E-45	72%	myb-like_DNA-binding_protein_FlbD	Fusarium_oxysporum_f._sp._vasinfectum_25433
475668598	10-109	24%	1.00E-45	73%	Myb-like_DNA-binding_protein_myb-1	Fusarium_oxysporum_f._sp._cubense_race_4
590042295	10-109	24%	1.00E-45	73%	myb-like_DNA-binding_protein_FlbD	Fusarium_oxysporum_f._sp._melonis_26406
587681895	1-135	24%	1.00E-45	73%	hypothetical_protein_FOZG_17811	Fusarium_oxysporum_Fo47

342879982	10-109	24%	1.00E-45	73%	hypothetical_protein_FOXB_08245	Fusarium_oxysporum_Fo5176
587678496	10-109	24%	1.00E-45	73%	myb-like_DNA-binding_protein_FlbD	Fusarium_oxysporum_FOSC_3-a
115388409	4-109	26%	2.00E-45	74%	hypothetical_protein_ATEG_02532	Aspergillus_terreus_NIH2624
119189513	4-109	26%	2.00E-45	75%	hypothetical_protein_CIMG_04804	Coccidioides_immitis_RS
584415425	4-109	26%	2.00E-45	73%	Homeodomain-like	Penicillium_roqueforti
607886137	4-109	26%	2.00E-45	71%	hypothetical_protein_H101_07912_partial	Trichophyton_interdigitale_H6
46110066	8-108	25%	2.00E-45	74%	hypothetical_protein_FG01915.1	Fusarium_graminearum_PH-1
563288574	5-105	25%	2.00E-45	76%	hypothetical_protein_SBOR_10075	Sclerotinia_borealis_F-4157
453087618	1-102	25%	2.00E-45	76%	hypothetical_protein_SEPMUDRAFT_147483	Sphaerulina_musiva_SO2202
50551263	4-115	27%	3.00E-45	71%	YALI0D21230p	Yarrowia_lipolytica
409191721	4-109	26%	3.00E-45	72%	putative_FlbD	Penicillium_digitatum
425775264	4-109	26%	3.00E-45	72%	MYB_family_conidiophore_development_protein_FlbD_putative	Penicillium_digitatum_PHI26
633050508	4-109	26%	3.00E-45	71%	hypothetical_protein_H109_01718	Trichophyton_interdigitale_MR816
327296946	4-109	26%	3.00E-45	71%	hypothetical_protein_TERG_06164	Trichophyton_rubrum_CBS_118892
326476334	4-109	26%	3.00E-45	71%	MYB_family_conidiophore_development_protein_FlbD	Trichophyton_tonsurans_CBS_112818
260949153	13-116	25%	3.00E-45	73%	hypothetical_protein_CLUG_00032	Clavispora_lusitaniae_ATCC_42720
150864981	69-172	25%	3.00E-45	72%	hypothetical_protein_PICST_67483	Scheffersomyces_stipitis_CBS_6054
302511417	4-109	26%	4.00E-45	71%	MYB_family_conidiophore_development_protein_FlbD_putative	Arthroderma_benhamiae_CBS_112371
302662807	4-109	26%	4.00E-45	71%	MYB_family_conidiophore_development_protein_FlbD_putative	Trichophyton_verrucosum_HKI_0517
296811476	4-109	26%	5.00E-45	71%	FlbD	Arthroderma_otae_CBS_113480
525581812	4-109	26%	5.00E-45	72%	hypothetical_protein_PDE_03008	Penicillium_oxalicum_114-2
629715278	5-105	25%	5.00E-45	74%	trichome_differentiation_protein_GL1	Metarhizium_anisopliae_ARSEF_23
629694995	5-105	25%	7.00E-45	74%	trichome_differentiation_protein_GL1	Metarhizium_acridum_CQMa_102
238483391	4-109	26%	1.00E-44	71%	MYB_family_conidiophore_development_protein_FlbD_putative	Aspergillus_flavus_NRRL3357
358366824	4-109	26%	1.00E-44	72%	MYB_family_conidiophore_development_protein_FlbD	Aspergillus_kawachii_IFO_4308
145229201	4-109	26%	1.00E-44	72%	MYB_family_conidiophore_development_protein_FlbD	Aspergillus_niger_CBS_513.88
169766640	4-109	26%	1.00E-44	71%	MYB_family_conidiophore_development_protein_FlbD	Aspergillus_oryzae_RIB40
326481196	4-109	26%	1.00E-44	71%	MYB_family_conidiophore_development_protein_FlbD	Trichophyton_equinum_CBS_127.97
315044197	4-109	26%	2.00E-44	71%	hypothetical_protein_MGYG_06020	Arthroderma_gypseum_CBS_118893
565933435	6-110	26%	3.00E-44	73%	hypothetical_protein_G647_06777	Cladophialophora_carrionii_CBS_160.54
531862815	29-132	25%	3.00E-44	71%	trichome_differentiation_protein_GL1	Ophiocordyceps_sinensis_CO18
632918574	8-107	24%	3.00E-44	74%	trichome_differentiation_protein_GL1	Villosiclava_virens
628238585	6-110	26%	4.00E-44	73%	hypothetical_protein_A1O1_04095	Capronia_coronata_CBS_617.96
295663521	4-109	26%	4.00E-44	72%	MYB_family_conidiophore_development_protein_FlbD	Paracoccidioides_sp._lutzii_Pb01
628345292	6-136	32%	7.00E-44	64%	myb-like_DNA-binding_protein_FlbD	Cladophialophora_psammophila_CBS_110553

225562948	4-109	26%	7.00E-44	72%	conserved_hypothetical_protein	Ajellomyces_capsulatus_G186AR
628257767	6-109	25%	7.00E-44	73%	myb-like_DNA-binding_protein_FlbD	Capronia_epimyces_CBS_606.96
225677799	4-109	26%	9.00E-44	72%	myb-like_DNA-binding_protein_partial	Paracoccidioides_brasiliensis_Pb03
327355851	4-109	26%	1.00E-43	72%	MYB_family_conidiophore_development_protein_FlbD	Ajellomyces_dermatitidis_ATCC_18188
261196364	4-109	26%	1.00E-43	72%	MYB_family_conidiophore_development_protein_FlbD	Ajellomyces_dermatitidis_SLH14081
226287464	4-109	26%	1.00E-43	72%	MYB_family_conidiophore_development_protein_FlbD	Paracoccidioides_brasiliensis_Pb18
440638474	1-104	25%	1.00E-43	70%	hypothetical_protein_GMDG_03182	Pseudogymnoascus_destructans_20631-21
582959665	4-114	27%	2.00E-43	70%	hypothetical_protein_DRE_02746	Drechslerella_stenobrocha_248
240279771	6-109	25%	2.00E-43	72%	MYB_family_conidiophore_development_protein_FlbD	Ajellomyces_capsulatus_H143
328350610	6-109	25%	2.00E-43	70%	Myb_protein	Komagataella_pastoris_CBS_7435
254566205	17-120	25%	2.00E-43	70%	Myb-like_DNA-binding_protein	Komagataella_pastoris_GS115
531980099	86-191	26%	3.00E-43	72%	hypothetical_protein_BDFG_06839	Ajellomyces_dermatitidis_ATCC_26199
190345090	19-130	27%	4.00E-43	67%	hypothetical_protein_PGUG_01009	Meyerozyma_guilliermondii_ATCC_6260
146423407	19-130	27%	5.00E-43	67%	hypothetical_protein_PGUG_01009	Meyerozyma_guilliermondii_ATCC_6260
598068077	77-183	26%	5.00E-43	69%	hypothetical_protein_SPAPADRAFT_71207	Spathaspora_passalidarum_NRRL_Y-27907
149248172	179-282	25%	8.00E-43	70%	hypothetical_protein_LELG_00993	Lodderomyces_elongisporus_NRRL_YB-4239
406602479	56-189	33%	1.00E-42	60%	Myb-like_DNA-binding_protein_BAS1	Wickerhamomyces_ciferrii
448091581	133-236	25%	1.00E-42	70%	Piso0_004615	Millerozyma_farinosa_CBS_7064
67515995	4-250	62%	7.00E-42	43%	hypothetical_protein_AN0279.2	Aspergillus_nidulans_FGSC_A4
448512344	157-260	25%	9.00E-42	68%	hypothetical_proteinelix-loop-helix_(HLH)_transcription_factor	Candida_orthopsilosis_Co_90-125
45190431	4-109	26%	3.00E-41	64%	AEL176Cp	Ashbya_gossypii_ATCC_10895
513032193	4-109	26%	3.00E-41	64%	AaceriAEL176Cp	Saccharomycetaceae_sp._Ashbya_aceri
363754559	4-110	26%	4.00E-41	63%	hypothetical_protein_Ecym_6298	Eremothecium_cymbalariae_DBVPG*7215
241951530	111-215	26%	1.00E-40	65%	myb-like_transcription_factor_putative	Candida_dubliniensis_CD36
629674196	3-109	26%	1.00E-40	62%	putative_myb_family_conidiophore_development_protein	Eutypa_lata_UCREL1
584394928	10-113	25%	2.00E-40	67%	unnamed_protein_product	Kuraishia_capsulata_CBS_1993
459373492	133-237	26%	4.00E-40	67%	hypothetical_protein_G210_1739_partial	Candida_maltosa_Xu316
294656110	87-190	25%	4.00E-40	66%	DEHA2C15378p	Debaryomyces_hansenii_CBS767
68486563	113-218	26%	8.00E-40	64%	hypothetical_protein_CaO19.14163	Candida_albicans_SC5314
399166941	18-130	28%	9.00E-40	65%	probable_regulator_of_conidiation_rca-1	Claviceps_purpurea_20.1
238882331	113-218	26%	9.00E-40	64%	conserved_hypothetical_protein	Candida_albicans_WO-1
354546598	159-259	25%	1.00E-39	68%	hypothetical_protein_CPAR2_209750	Candida_parapsilosis
255724626	140-245	26%	4.00E-39	65%	conserved_hypothetical_protein	Candida_tropicalis_MYA-3404
320588865	3-125	31%	5.00E-39	59%	myb-like_DNA-binding_protein	Grosmannia_clavigera_kw1407
255720094	6-110	25%	1.00E-38	64%	KLTH0H10494p	Lachancea_thermotolerans

562975059	22-172	33%	3.00E-37	53%	Myb-like_DNA-binding_protein_myb-1	Ogataea_parapolymorpha_DL-1
85102824	4-115	27%	3.00E-36	60%	hypothetical_protein_NCU01312	Neurospora_crassa_OR74A
2253310	7-115	27%	4.00E-36	59%	MYB-1	Neurospora_crassa
336266786	4-115	27%	5.00E-36	59%	regulator_of_conidiation	Sordaria_macrospora_k-hell
336472607	4-115	27%	8.00E-36	59%	hypothetical_protein_NEUTE1DRAFT_76192	Neurospora_tetrasperma_FGSC_2508
121703107	4-227	61%	1.00E-35	41%	MYB_family_conidiophore_development_protein_FlbD_putative	Aspergillus_clavatus_NRRL_1
576046128	11-114	25%	1.00E-34	62%	hypothetical_protein_CTHT_0066400	Chaetomium_thermophilum_var._thermophilum_DSM_1495
154280264	4-93	26%	1.00E-32	61%	conserved_hypothetical_protein	Ajellomyces_capsulatus_NAm1
320033311	4-73	17%	1.00E-31	84%	MYB_family_conidiophore_development_protein_FlbD	Coccidioides_posadasii_str._Silveira
327296950	4-73	17%	3.00E-31	79%	hypothetical_protein_TERG_06165	Trichophyton_rubrum_CBS_118892
399163653	55-167	28%	8.00E-30	54%	RCA-1_regulator_of_conidiation	Claviceps_purpurea_20.1
385881328	14-118	25%	2.00E-29	56%	Myb-like_transcription_regulator	Magnaporthe_oryzae
389623951	88-192	25%	2.00E-29	56%	hypothetical_protein_MGG_06898	Magnaporthe_oryzae_70-15
399163665	19-126	26%	2.00E-28	52%	RCA-1_regulator_of_conidiation	Claviceps_purpurea_20.1
50306551	60-175	28%	1.00E-27	48%	hypothetical_protein	Kluyveromyces_lactis_NRRL_Y-1140
399163654	52-154	25%	1.00E-27	50%	probable_regulator_of_conidiation_rca-1	Claviceps_purpurea_20.1
358401247	7-108	25%	4.00E-27	54%	hypothetical_protein_TRIATDRAFT_53194_partial	Trichoderma_atroviride_IMI_206040
574141120	81-217	34%	5.00E-27	43%	conserved_hypothetical_protein	Kluyveromyces_marxianus_DMKU3-1042
472585203	60-167	26%	3.00E-26	52%	transcriptional_activator_Myb	Rhodosporidium_toruloides_NP11
367024213	12-115	26%	8.00E-26	55%	hypothetical_protein_MYCTH_2300719	Myceliophthora_thermophila_ATCC_42464
572274422	9-109	25%	1.00E-25	52%	myb_superfamily_protein_partial	Trichoderma_reesei_RUT_C-30
589114073	1-100	24%	2.00E-25	53%	predicted_protein	Trichoderma_reesei_QM6a
358389843	1-100	24%	2.00E-25	54%	hypothetical_protein_TRIVIDRAFT_19767_partial	Trichoderma_virens_Gv29-8
116206630	12-118	26%	1.00E-24	51%	hypothetical_protein_CHGG_02608	Chaetomium_globosum_CBS_148.51
512193723	12-128	26%	2.00E-24	50%	myb_family_conidiophore_development_protein	Ophiostoma_piceae_UAMH_11346
367036989	12-115	26%	2.00E-24	54%	hypothetical_protein_THITE_2106830	Thielavia_terrestris_NRRL_8126
634354509	118-222	25%	2.00E-24	52%	hypothetical_protein_MVLG_00426	Microbotryum_violaceum_p1A1_Lamole
